# Quantitative examination of video-recorded NHS Health Checks: comparison of the use of QRISK2 versus JBS3 cardiovascular risk calculators

**DOI:** 10.1136/bmjopen-2020-037790

**Published:** 2020-09-25

**Authors:** Christopher J Gidlow, Naomi J Ellis, Lisa Cowap, Victoria A Riley, Diane Crone, Elizabeth Cottrell, Sarah Grogan, Ruth Chambers, David Clark-Carter

**Affiliations:** 1 Centre for Health and Development, Staffordshire University, Stoke-on-Trent, UK; 2 Department of Psychology, Staffordshire University, Stoke on Trent, UK; 3 Cardiff School of Sport and Health Sciences, Cardiff Metropolitan University, Cardiff, UK; 4 School of Primary, Community and Social Care, Keele University, Staffordshire, UK; 5 Department of Psychology, Manchester Metropolitan University, Manchester, UK; 6 Stoke-on-Trent Clinical Commissioning Group, Stoke on Trent, UK

**Keywords:** preventive medicine, primary care, coronary heart disease

## Abstract

**Objectives:**

Quantitatively examine the content of National Health Service Health Check (NHSHC), patient–practitioner communication balance and differences when using QRISK2 versus JBS3 cardiovascular disease (CVD) risk calculators.

**Design:**

RIsk COmmunication in NHSHC was a qualitative study with quantitative process evaluation, comparing NHSHC using QRISK2 or JBS3. We present data from the quantitative process evaluation.

**Setting and participants:**

Twelve general practices in the West Midlands (England) conducted NHSHC using JBS3 or QRISK2 (6/group). Patients were eligible for NHSHC based on national criteria (aged 40–74, no existing cardiovascular-related diagnoses, not taking statins). Recruitment was stratified by patients’ age, gender and ethnicity.

**Methods:**

Video recordings of NHSHC were coded, second-by-second, to quantify who was speaking and what was being discussed. Outcomes included consultation duration, practitioner verbal dominance (ratio of practitioner:patient speaking time (pr:pt ratio)) and proportion of time discussing CVD risk, risk factors and risk management.

**Results:**

173 video-recorded NHSHC were analysed (73 QRISK, 100 JBS3). The sample was 51% women, 83% white British, with approximately equal proportions across age groups. NHSHC duration varied greatly (6.8–38.0 min). Most (60%) lasted less than 20 min. On average, CVD risk was discussed for less than 2 min (9.06%±4.30% of consultation time). There were indications that, compared with NHSHC using JBS3, those with QRISK2 involved less CVD risk discussion (JBS3 M=10.24%, CI: 8.01–12.48 vs QRISK2 M=7.44%, CI: 5.29–9.58) and were more verbally dominated by practitioners (pr:pt ratio JBS3 M=3.21%, CI: 2.44–3.97 vs QRISK2=2.35%, CI: 1.89–2.81). The largest proportion of NHSHC time was spent discussing causal risk factors (M=37.54%, CI: 32.92–42.17).

**Conclusions:**

There was wide variation in NHSHC duration. Many were short and practitioner-dominated, with little time discussing CVD risk. JBS3 appears to extend CVD risk discussion and patient contribution. Qualitative examination of how it is used is necessary to fully understand the potential benefits of these differences.

**Trial registration number:**

ISRCTN10443908.

**Strengths and limitations of this study**

- Video recordings of National Health Service Health Check provided an objective record of practice across a range of general practices.
- Second-by-second coding was used to characterise the content of health checks that used either QRISK2 or JBS3 cardiovascular disease risk calculators.
- The study was limited to 12 general practices in the West Midlands of England.
- Initial target sample size was not achieved.
- Qualitative data are necessary to provide further insight.

## Introduction

Cardiovascular disease (CVD) accounts for over a quarter of UK deaths and costs the National Health Service (NHS) around £9 billion annually.^1^ As CVD mortality decreases in the UK, there is a high prevalence of people living with CVD.^2^ Prevention, therefore, remains a priority, for which the NHS Health Check (NHSHC) Programme is an important part.^3 4^ NHSHC aims to assess CVD risk factors among adults in England aged 40–74 years who are not known to have certain cardiovascular-related diseases.^5^ It is the largest CVD risk identification and management programme of its kind globally, and has been linked with some increases in the detection of risk factors and chronic disease, and statin prescriptions,^6 7^ with mixed predictions of benefits from microsimulation studies.^8 9^

NHSHC consultations typically take place in primary care with a primary care nurse, and comprise: (a) assessing patients’ CVD risk, (b) communication of CVD risk, which should inform (c) discussion of CVD risk management through lifestyle or subsequent medical appointments or referrals. The NHSHC competence framework specifies that practitioners should understand CVD risk and be able to communicate to patients their CVD risk such that ‘the patient understands their level of risk’ (Public Health England,^10^ p21). This should lead to discussion with the patient about management of that risk through ‘person-centred conversations about their own reasons for change’ (Public Health England,^10^ p21), using techniques like motivational interviewing. This accords with the idea of shared decision-making, described as the pinnacle of patient-centred care (Barry and Edgman-Levitan).^11^ In shared decision making, health professionals and individuals work together to agree management or support, based on evidence and the patient’s preferences.^12^

To date, little is known about the nature or content of real-world NHSHC consultations. Evidence regarding what takes place in the consultations is mainly limited to qualitative data from retrospective interviews with patients and practitioners asked to recall and reflect on their experiences.^13^ Such data do have value. However, they do not present a complete understanding of the dynamics and interactions that can initiate subsequent actions or interventions (eg, referral of patients to effective lifestyle-support programmes, appropriate specialist referrals) that could lead to improved patient outcomes.

This paper reports the relative contributions of practitioners and patients, and the time spent discussing CVD risk and risk management in NHSHC consultations. This is important to understand as lack of time is the barrier to shared decision-making, most frequently cited by practitioners and patients.^14^ Consultations in which the health professional and patient work together to identify risk-management strategies, taking evidence and patient preference into account,^12^ require more time than didactic encounters involving little patient involvement.^14^ Studies of clinician–patient interactions have identified that short, clinician-dominated (or ‘paternalistic’) consultations, are less patient-centred and linked with low patient and clinician satisfaction,^15–18^ which in turn, have been linked with poorer patient outcomes, such as adherence to clinical recommendations and health-promoting behaviour.^19 20^ While there is some evidence that brief interventions of just a few minutes can contribute towards behavioural change,^21^ motivational interviewing, a technique recommended in NHSHC, may require 15 min or more to be effective.^22^ Pieterse *et al*^14^ summarised the relatively modest literature that specifically links time with shared decision-making. The authors noted that health professionals often feel under time pressure. Time with patients is constrained by a schedule that determines the maximum appointment length, and the need to complete all clinical and administrative tasks. Hurried practitioners might interrupt the conversation^23^ or present information too quickly or use inappropriate language, reducing the likelihood that information will be retained.^14^ For NHSHC, it is likely to be important that time is sufficient for a two-way interaction in which patients can be supported to develop their own CVD risk-management strategy.

Time is also likely to be an important consideration in communication of CVD risk in NHSHC. Cypher identified prerequisites of shared decision-making that included ‘accurate, impartial and comprehensible information’ presented by a practitioner who is ‘proficient in communication and able to individualize data to a particular situation’ (Cypher,^24^ p1). All NHSHCs must involve assessment of CVD risk using QRISK (QRISK2 and, more recently, QRISK3).^25 26^ QRISK, which is embedded within primary care medical record software, provides a percentage risk of a CVD event in the next 10 years. This has to be communicated to patients for the NHSHC to be considered ‘complete’. It is integrated within the general practice electronic medical record software, so it can be calculated from pre-populated and new data. The score is then directly saved into the patient’s record. However, there are limitations with the QRISK score and how it is used. First, 10-year risk estimates, such as those presented by QRISK, have been criticised for being heavily influenced by age and gender, thereby underestimating risk in younger adults and women, and not accounting for risk from other diseases as effectively as long-term (lifetime) estimates.^27 28^ Second, qualitative studies indicate limited practitioner and patient understanding of percentage CVD risk^13^ and that practitioners report difficulties in explaining percentage CVD risk.^29–32^ In turn, patients attending NHSHCs have been unable to recall being provided with a risk score or find it confusing.^13^

In 2014, the Joint British Societies recommendations on the prevention of CVD (JBS3) launched the JBS3 risk calculator. JBS3 has a primary focus on lifetime risk,^27^ includes heart age^33–35^ and uses multiple visual displays to present risk (eg, Cates plot, image of a heart for heart age, visual analogue scales).^36^ It has also been designed to support communication of tailored information by allowing manipulation and, thus, demonstration of the effects of risk factor modification (eg, smoking cessation) on lifetime risk trajectory. There is some evidence that lifetime risk, as provided by JBS3, can identify raised CVD risk in some people that would not be picked up through conventional 10-year risk estimates (given within QRISK2);^37^ that heart age is more easily communicated to, and recalled by, patients;^38 39^ and that graphical displays can be preferable for promoting risk-reducing behaviour.^40^ Collectively, these attributes of JBS3 might accommodate a more extensive and higher quality interaction between patients and practitioners.^33 36^ However, a comparison of the relative benefits JBS3 and QRISK2 for communicating CVD risk in NHSHC has not been undertaken. Given that practitioners currently have choices in CVD risk calculators, establishing their relative values is important, particularly with regards to knowing which approach best promotes communication that supports positive behavioural change.

In summary, NHSHC aims to assess CVD risk and to prompt minimisation of risks identified. To date, there is insufficient knowledge about how NHSHCs are conducted, the time spent discussing CVD risk and its management, and the potential of alternative CVD risk calculators, like JBS3. RIsk COmmunication (RICO) in NHSHC is a large study of practitioner and patient perceptions and understanding of CVD risk when using the JBS3 or QRISK2 CVD risk calculators.^41^ Video-recording methods in RICO have provided the first objective data of real-world NHSHC, allowing for extensive qualitative and quantitative analysis. In this paper we present the quantitative data, with three aims: (a) examine the time spent discussing CVD risk, risk factors and management in NHSHC consultations; (b) explore the level of patient–practitioner communication balance; (c) compare a and b when NHSHCs are conducted using QRISK2 versus JBS3 CVD risk calculators.

## Method

### Study design

The RICO Study is a qualitative study with quantitative process evaluation. A detailed description of the overall study, including sampling, recruitment and data collection is available.^41^ Here, we report a quantitative comparison of the content of video-recorded NHSHC consultations.

### Patient and public involvement and Engagement

Our approach was informed by extensive patient and public involvement and engagement (PPIE). Engagement with patient participation groups at three general practices on two occasions was used to gather opinion on the study concept and overall design, and subsequently, the methods and protocols, participants’ consent and debrief processes, and for the development of the coding framework. Four mock NHSHCs were used to test protocols, such as camera placement and video recording. For ongoing involvement of patients, two patient representatives sat on the Study Steering Committee and a virtual study patient group was established using a closed Facebook group (>260 members).

### Setting and general practice recruitment

Data were collected across 12 general practices in the West Midlands of England that already delivered NHSHC (January 2017–February 2019). Practices were recruited via the local Clinical Research Network. Six practice pairs, approximately matched by deprivation, were randomly assigned to one of two groups: QRISK2 (usual practice)—practitioners continue to use QRISK2 to calculate CVD risk; JBS3 (intervention)—practitioners calculate the CVD risk using JBS3 following brief training about the platform (no training regarding risk communication was provided). To enable assessment of differences arising from JBS3 compared with QRISK2, as a minimum, those using JBS3 were asked to use the output screens showing: (a) heart age—estimate of heart age compared with someone of the same gender, ethnicity and risk factors at optimal levels; (b) healthy years—estimate of the age the patient can expect to reach without a CVD event (or event-free survival age). They were also asked to show patients the effect of intervention on one or more of the CVD risk metrics (eg, effect of smoking cessation on event-free survival age). A brief training video was created to guide clinicians (https://www.youtube.com/watch?v=idecGzlwIc4&feature=youtu.be).

### Participants and recruitment

Patients were those eligible for NHSHC based on national criteria. Thus people were excluded if they were outside of the target age range (40–74 years), had existing diagnoses for certain cardiovascular-related chronic conditions, took statins, had received an NHSHC in the last 5 years or were known to be at high risk of CVD.^25^ Within each practice, the list of eligible patients was stratified according to age, gender and ethnicity, and invitations were sent to a representative sample. Postal invitations were distributed and follow-up calls were made by practice staff.

Practitioners all worked within primary care (nine healthcare assistants (HCA), five practice nurses, one sister) and already undertook NHSHC as part of routine practice. The only exception was one HCA who was new to NHSHC delivery.

### Data sources and processing

Participating practices were asked to video record NHSHCs using the allocated CVD risk calculator until 20 useable consultations were recorded (with written permission from patients and practice consent). Video-recorded NHSHC consultations were the main data source. Recorded NHSHCs were viewed by two authors (LC and VAR) and the content of consultations was characterised using a second-by-second coding framework developed specifically for this study. The framework comprised 36 items grouped into six categories: patient–practitioner communication, health check general (eg, collecting and inputting data), risk dialogue (eg, overall discussion of risk, 10-year risk score reference, heart age, patient question on CVD risk), causal CVD risk factors (medical, lifestyle), risk management (lifestyle intervention, medical intervention; online supplemental file 1). This allowed derivation of aggregate indicators for each consultation, to allow between-group comparisons for:

10.1136/bmjopen-2020-037790.supp1 Supplementary data

- Length of NHSHC.
- Time (absolute and proportion of consultation) discussing CVD risk, CVD risk factors (overall, lifestyle, medical) and risk management (lifestyle, medical).
- Practitioner dominance (ratio of practitioner:patient speaking time).
- Number and proportion of patients asking questions about CVD risk.
- Use of heart age, healthy years (event-free survival age) and risk score manipulation (as fidelity check in the JBS3 group).

As noted previously,^41^ the coding process and framework development was iterative, using four mock NHSHCs that were video recorded as part of PPIE. To reach consistency in approach, two authors (NJE and LC) coded mock NHSHCs by consensus. Author VAR then coded the same four consultations independently and Intraclass Correlation Coefficients (ICCs) demonstrated excellent inter-rater reliability (ICCs from 0.968 to 0.995). Once data collection had begun, NHSHC were coded by authors LC and VAR, with verification of 10% (2 in every 20 independently coded) to mitigate the risk of coder drift throughout the study. ICCs ranged from 0.992 to 0.999, indicating excellent inter-rater reliability.

Data were extracted from patients’ medical records on patients’ sex, age and ethnic background (classified as white British (WBRI) or black, Asian and minority ethnic (BAME)).

### Sample size

The target sample size was 240 (120 per group). A sample size calculation undertaken for the desired between-group quantitative comparison estimated that 120 consultations per group, six clusters per group with a two-tailed probability and alpha of 0.05, would provide statistical power of 0.8 to detect an effect size (r)=0.24 (small to medium effect). The overall number of eligible practices from which the clusters were chosen was 625 (based on number of general practices in the West Midlands).

### Statistical methods

Following checks for normal distributions, QRISK2 and JBS3 groups were compared according to key variables. Confidence intervals (95% CIs) were calculated taking account of the nature of the sampling which was in clusters; as usual, where the CIs of the two groups did not overlap, the groups were considered to differ significantly. Data processing and analysis were performed in SPSS V.26.

## Results

### Participants

One hundred and seventy-five video-recorded NHSHCs were completed, of which 173 were included in analysis (QRISK=73; JBS3=100; 2 excluded for practitioner process error that invalidated the consultation). The sample comprised approximately equivalent proportions of men and women, and proportions in WBRI versus BAME groups that were representative of the geographical region (table 1). Average age of the JBS3 group (M=60.87; CI: 58.91–62.83) was higher than QRISK2 (M=54.70, CI: 51.66–57.70), while mean 10-year CVD risk was slightly higher in the JBS3 group (M=9.71, CI: 7.85–11.57) than the QRISK2 group (M=8.69, CI: 5.56–11.81).

**Table 1:** Participants’ characteristics

		Total		JBS3		QRISK		Difference
		n	%	n	%	n	%	r *
Age (years)	40–54	60	34.68	24	24.00	36	49.32	0.32
	55–64	54	31.21	30	30.00	24	32.88	
	65–74	59	34.10	46	46.00	13	17.81	
	Total	173		100		73		
Gender	Male	86	49.71	49	49.00	37	50.68	0.05
	Female	87	50.29	51	51.00	36	49.32	
	Total	173		100		73		
Ethnicity†	WBRI	144	83.24	81	81.00	63	86.30	0.07
	BAME groups	29	16.76	19	19.00	10	13.70	
	Total	173		100		73		
CVD risk category‡	Low	104	60.12	57	57.00	47	64.38	0.06
	Medium–high	67	38.73	41	41.00	26	35.62	
	Total	171		98		73		

*Effect size, r: where 0.1 is small, 0.3 is medium, 0.5 is large.

†Ethnicity: White British (WBRI); black, Asian and minority ethnic group (BAME).

‡Cardiovascular disease (CVD) risk categories: low is 10%, medium-high is ≥10%.

### Length of NHSHC consultations

**Figure 1:**
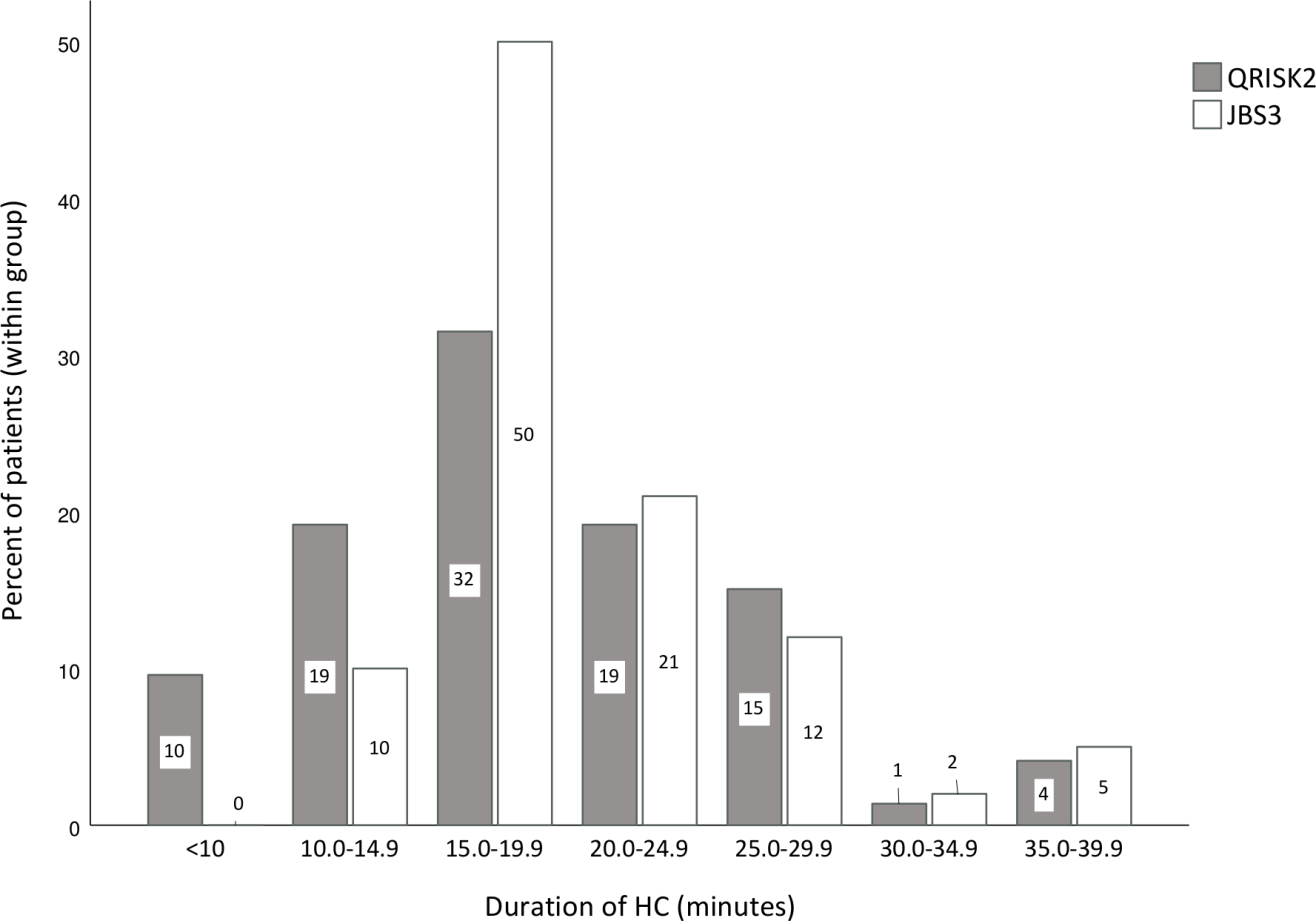
Duration of NHSHC consultation by CVD risk calculator group. HC, Health Check.

Table 2 summarises the characteristics of NHSHC consultations by group and overall. There was a wide range in consultation length, from just 6.8 to over 38 min, but the majority were between 15 and 20 min (figure 1). Consultations were only slightly shorter on average in the QRISK2 compared with JBS3 group (with a relatively small effect size of 0.13).

**Table 2:** Between-group comparisons of characteristics of NHSHC consultation

	Total		JBS3		QRISK		Difference* (QRISK vs JBS3)
	Mean (95% CI)	SD	Mean (95% CI)	SD	Mean (95% CI)	SD	r
**Duration** (min)	20.06 (18.87–21.24)	6.21	20.66 (18.89–22.42)	5.65	19.24 (15.28–23.19)	6.84	0.13
**Verbal dominance**							
% of total time practitioner speaking	50.07 (45.90–54.24)	9.55	46.60 (41.36–51.84)	8.79	54.82 (50.00–59.64)	8.48	0.42
% of total time patient speaking	23.37 (19.87–26.87)	10.62	24.67 (20.47–28.87)	10.53	21.6 (15.71–27.43)	10.56	0.15
Verbal dominance ratio †	2.70 (2.23–3.19)	1.50	2.35 (1.89–2.81)	1.31	3.21 (2.44–3.97)	1.62	0.27
**% of total HC time**:							
Discussing CVD risk	9.06 (7.36–10.76)	4.30	10.24 (8.01–12.48)	4.07	7.44 (5.29–9.58)	4.08	−0.32
Discussing risk factors							
Total	37.54 (32.92–42.17)	12.96	35.33 (27.76–42.90)	13.29	40.58 (36.20–44.96)	11.91	0.22
Medical ‡	21.34 (18.35–24.33)	9.41	20.13 (15.33–24.94)	9.38	22.98 (19.96–26.31)	9.26	0.16
Lifestyle §	16.11 (13.79–18.44)	7.03	15.08 (11.87–18.30)	6.49	17.52 (14.16–20.88)	7.53	0.16
Discussing risk management							
Total	19.64 (16.48–22.81)	11.37	18.82 (13.92–23.73)	11.12	20.77 (16.59–24.94)	11.69	0.08
Lifestyle	16.59 (13.44–19.74)	10.20	15.94 (11.27–20.62)	10.04	17.48 (12.95–22.01)	10.41	0.08
Medical¶	63%		58%		69.9%		−0.12

*Effect size, r: where 0.1 is small, 0.3 is medium, 0.5 is large.

†Ratio of practitioner: patient talking (where higher value indicates greater practitioner verbal dominance).

‡Includes medical history, family history, weight, blood pressure, cholesterol, diabetes, mental health and well-being.

§Includes diet, alcohol, physical activity, smoking.

¶Percentage of patients with whom medical interventions were discussed.

CVD, cardiovascular disease; HC, health check; NHSHC, National Health Service Health Check.

### Discussion of CVD risk

Overall, less than 10% of overall consultation time was devoted to CVD risk discussion, which equated to 1.7±0.83 min. A higher proportion of consultation time was spent discussing CVD risk using JBS3 (equivalent to 2.1±0.82 min) compared with QRISK2 (equivalent to 1.31±0.63 min), with a medium effect size. Nearly all NHSHCs in both groups included reference to the 10-year percentage CVD risk score (94% vs 94.5%, r=0.01). The proportion of patients asking questions about CVD risk was higher in the JBS3 versus QRISK2 group (32.0% vs 12.3%, r=0.23).

Within the JBS3 group, nearly all practitioners discussed heart age (100%) and healthy years (97%), and manipulated the risk score(s) to show the potential effect of intervention on risk (92%). This showed fidelity to the requested minimum use of JBS3 outputs. The use of heart age and risk manipulation was also evident in 52.1% and 21.9% of QRISK2 consultations, respectively. This is a result of two general practices in the QRISK2 group using Informatica (a software addition that offers some JBS3 functionalities), and because heart age and allowing manipulation are possible (but not main features) in QRISK2.

### Discussion of CVD risk factors and risk management

Over one-third of total NHSHC time was spent discussing CVD risk factors. This was slightly higher in the QRISK2 versus JBS3-informed NHSHCs, but with wide variation within groups (table 2).

Interventions to manage risk were discussed for approximately one-fifth of total consultation time and predominantly related to lifestyle, rather than medical intervention, which was not discussed at all in over 30% of QRISK2 and 42% of JBS3-informed NHSHCs (r=−0.12).

### Verbal dominance

**Figure 2:**
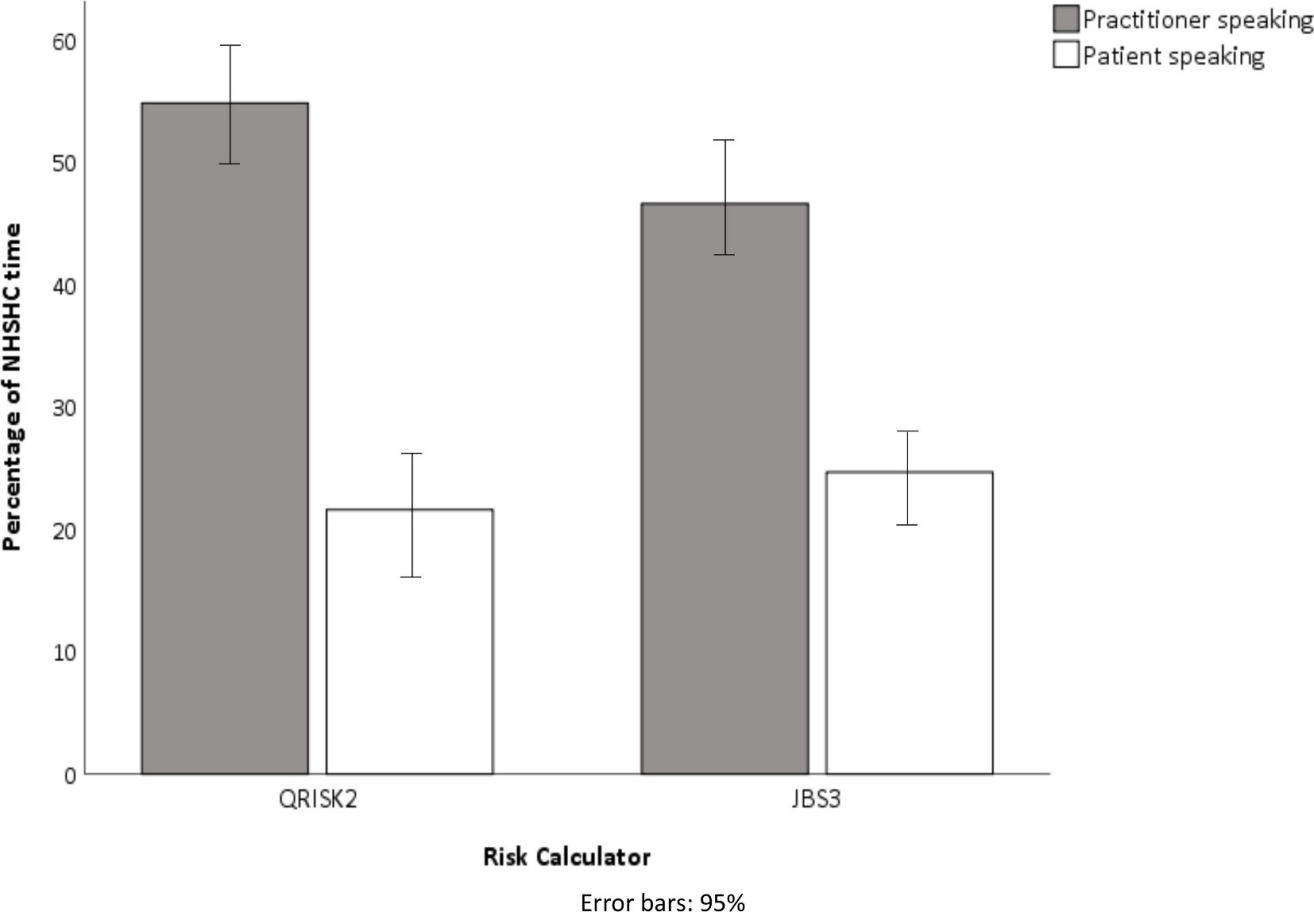
Mean percentage of total NHSHC time with speaking by practitioner or patient. NHSHC, NHS Health Check.

Practitioners spoke for just over half of total time in QRISK2 consultations and just under half in JBS3 (figure 2). There was an indication of higher practitioner verbal dominance in NHSHC using QRISK2 versus JBS3 (r=0.27).

## Discussion

### Main findings

We present the first objective data on the content of NHSHC consultations, with comparison of QRISK2 and JBS3 CVD risk calculators. Second-by-second coding of 173 video-recorded NHSHC consultations from 12 general practices provided new insights regarding duration, verbal dominance and allocation of time to different components of the consultation. Main findings in relation to our study aims were as follows. First, the length of NHSHC consultations was varied and often short (most <20 min). Less than 10% of time (<2 min) was spent discussing the individual’s calculated CVD risk, and the largest component was discussion of causal CVD risk factors. Second, there was evidence of practitioner verbal dominance as, on average, practitioners spoke for half of total consultation time (compared with ~23% with patients speaking). Third, compared with QRISK2, there were indications that use of JBS3 was associated with more discussion of CVD risk, less discussion of CVD risk factors, reduced practitioner verbal dominance and, on average, required only 1.4 additional minutes (<90 s).

### What this study adds

The variable and often short duration of NHSHC, combined with practitioner verbal dominance are a potential concern. Sixty per cent of NHSHC in the RICO study lasted less than 20 min, 18% less than 15 min and 4% were under 10 min. Nearly all (95%) had a verbal dominance ratio greater than 1, 57% had a ratio of 2 or more, and 36% were over 3, which indicates some degree of verbal dominance. Practitioner verbal dominance suggests more information provision than the desired patient-centred two-way interaction that seeks to understand the patients’ contexts, priorities and preferences, to allow appropriate goal-setting. While information provision is an important part of the process, the literature on clinician–patient interactions suggests that short, paternalistic primary care consultations are associated with low patient and clinician satisfaction.^18^ This can have negative implications for patient outcomes, potentially reducing adherence to clinician recommendations, and their ability to self-care or make informed decisions about their health.^15–17^

Practitioner–patient interactions are complex.^42^ The practitioner–patient balance has been discussed as a spectrum of locus of control, with a paternalistic health professional at one end and the informed patient at the other.^43^ The latter requires that control is mutual or exchanged, allowing a negotiated plan; in this case, a negotiated plan to manage CVD risk. The nature of interactions has been conceptualised and measured variously. For example, patient enablement, whereby patients have understanding, confidence and coping ability following enabling consultations,^15^ and physician–patient collaboration, where both physician and patient are active participants in consultations.^44^ A common feature of consultations that foster these beneficial relationships (and should lead to better outcomes) is the active participation of patients in the consultation and decisions about their healthcare. Our data indicate that there is scope in NHSHC for greater patient participation, especially as motivational interviewing should be a key feature of NHSHC and relies on a patient-centred approach.^25^

The length of appointments that practices allocated to NHSHC ranged from 15 to 30 min. This showed variation in the time that general practices were willing to give to NHSHC, and the associated time constraints for practitioners delivering NHSHC in those practices. Yet, video recordings highlighted even greater variability in the actual duration of practitioner–patient interaction (6.8–38 min). The length of primary care nurse appointments varies by appointment type. NHSHCs require a number of clinical and administrative tasks, such as: CVD risk assessment involving measurement of (and entry of data for) weight, blood pressure and, sometimes, cholesterol through point-of-care testing; lifestyle assessment (physical activity, alcohol, diet); explaining to patients their CVD risk score(s) and what it means; patient-centred discussion of risk management to prompt risk-reducing behaviours. In light of evidence that shared decision-making takes time and techniques like motivational interviewing might require 15 or more min to be effective, it is reasonable to suggest that consultations lasting much less than 20 min are unlikely to achieve all of the above while allowing time for appropriate levels of patient participation as part of a mutual exchange.

A primary focus of the RICO study was CVD risk communication in NHSHC. To promote health-protective behaviours that reduce CVD risk, practitioners need to understand the risk information and be able to communicate it effectively such that patients leave the consultation with the knowledge and intention to act.^40^ The little time dedicated to CVD risk discussion (<10%), especially when using QRISK2, accords with findings from the 2017 systematic review of qualitative studies.^45^ The review authors identified patients’ ‘limited understanding of the risk score’ as a common theme. Some patients reported improved understanding of CVD risk following their NHSHC, but many did not recall being told their risk, found it confusing or had misinterpreted their risk score.

Our data confirmed that nearly all practitioners using JBS3 conveyed heart age, healthy years and used risk score manipulation (as requested). This appeared to result in a slightly higher proportion of total time spent discussing CVD risk in JBS3-informed consultations (although not reaching significance), but equated to less than one additional minute, on average. This raises the question of whether JBS3 improve patient engagement and understanding of their risk, or simply require more information provision. That one in three patients in JBS3 consultations asked questions about their CVD risk (32%), compared with one in eight QRISK2 patients (12%), suggests better engagement, but qualitative analysis is required to provide a more complete understanding. RICO did not include extensive practitioner training in use of JBS3. Rather, an introductory briefing with a supporting digital versatile disc (DVD) were used to maintain the ecological validity of exploring how practitioners would use JBS3 if made available in practice (not the effect of additional training, which is generally not available in practice).^46^ Such training might be required and general CVD risk communication training for NHSHC practitioners has shown some benefits for practitioner confidence and understanding.^46^

The apparent focus on causal risk factors, discussion of which comprised 38% of NHSHC time overall (over 40% when using QRISK2), is perhaps an indication that practitioners did not spend much time explaining and inviting discussion around the risk score(s) and what they mean, but focused more on the potential causes and their management. This could be interpreted positively as a solution-focused approach that focuses on the tangible factors that might be easily understood; that is, how their medical history and lifestyle can lead and help to prevent CVD. However, it is important that this discussion is in the context of the patient’s CVD risk and tailored to their lifestyle and priorities. Risk communication is challenging.^36^ If delivered effectively, it can enhance knowledge and decision-making about treatment, and can empower and create autonomy.^47^ Conversely, poor communication of risk can cause patients anxiety and reduce confidence in health professionals, or may result in the perception that action is futile.^48^ Therefore, the 38% and 20% of NHSHC time spent discussing CVD risk factors and management, respectively, could be undermined if the patient is confused or alarmed by the preceding information about their CVD risk.

### Implications for practice

The 2019 green paper, *Advancing our health: prevention in the 2020s*, set out plans for an evidence-based review of NHSHC to maximise benefit in the next decade.^4^ This indicates a future for NHSHC, with evidence-based changes to delivery. Despite the importance of risk communication, associated recommendations in NHSHC’s best practice guidance^25^ and the practitioners’ competence framework that includes CVD risk communication,^10^ ours are the first objective data showing the varied and often short NHSHC, the limited time devoted to CVD risk discussion, and the potential for JBS3 to enhance this. We know that training in NHSHC is a challenge to optimal implementation.^13^ Practitioners generally receive little (or no) training in CVD risk communication for NHSHC and can lack the associated confidence and skills.^46^ Our data strengthen the case for NHSHC practitioner training, ideally with patient involvement, to redress the practitioner verbal dominance and help to make the discussion of CVD risk appropriate and positive for patients. JBS3 (or similar tools) could form part of this solution.

### Strengths and limitations

The strengths of this study include video recording of NHSHCs across a diverse range of practices stratified by deprivation, stratified sampling of patients and our comprehensive coding framework with excellent inter-rater reliability that offers a methodological contribution. A number of limitations are recognised. First, challenges with practice and patient recruitment meant that we did not achieve our target sample size of 240 (120/group). A larger sample size might have identified more marked between-group differences through increasing the precision of our estimates (ie, narrowing CIs). Second, there was a between-group difference in mean age. This did not translate into a difference in CVD risk, which would have introduced more systematic bias into the sample. But it would mean that risk was underestimated in the larger proportion of younger people in the QRISK2 group (compared with the JBS3 group). Third, these quantitative data do not tell us directly about quality of discussion or exact nature of patients’ response. However, the duration of time spent on different elements of risk communication and subsequent management planning do provide valuable indirect evidence about patient engagement, the balance of contributions and content within NHSHC consultations. Fourth, we cannot claim that our results are generalisable to the rest of England. But, we had a good balance of men/women, a good age range and proportions of WBRI and BAME groups that were appropriate for the region. Fifth, after commencing data collection, we discovered that two QRISK2 practices used Informatica, an addition to practice software that has some of the JBS3 functionalities. To maintain the ecological validity of a ‘usual care’ group, the 34 patients from these practices were retained, as they will be for qualitative analysis. Finally, it is possible that being video recorded affected practitioners’ behaviour (Hawthorne effect). To mitigate this, our PPIE explored camera position to best capture patients’ response and minimise practitioners’ awareness of the camera. Practitioners in mock NHSHCs reported forgetting about the camera during consultations. Moreover, the Hawthorne effect tends to improve performance,^49^ which would lead to an underestimate of issues with NHSHC delivery.

## Conclusions

Duration of the practitioner–patient interaction in NHSHC is varied and, in many cases, short. Our data highlight that little NHSHC time is devoted to discussing the patients’ calculated CVD risk, however it does appear that JBS3 can support an extended discussion and might prompt more patient engagement. The impact of practitioner verbal dominance on patient experience and outcomes should be further explored. Public Health England’s competence framework for NHSHC specifies the need for NHSHC practitioners to be trained in communicating the risk scores and to engage in person-centred conversations around risk-reducing behavioural changes.^10^ Although our qualitative findings will provide a more complete picture and elucidate the true potential of JBS3, our current data support the need for practitioners’ training in this area and, potentially, for additions to standard competencies within health professional training.

## Supplementary Material

Author's manuscript
