# Supplementary material for: Quantitative examination of video-recorded NHS Health Checks: comparison of the use of QRISK2 versus JBS3 cardiovascular risk calculators
Source: BMJ Open. 2020 Sep 25;10(9):e037790. doi: 10.1136/bmjopen-2020-037790 (PMC7520846; doi:10.1136/bmjopen-2020-037790)
Supplement: Supplementary data [file bmjopen-2020-037790supp001.pdf]

Table S1. Coding framework data variables

Category	Code	Code Title
Patient-practitioner communication	PrDT	Practitioner dominated talk
	PtDT	Patient dominated talk
	NT	No talking
Health Check general	I2HC	Introduction to Health Check
	HCSUM	Health Check summary
	C&ID	Collecting and Inputting Data
	TRG	Test result/recording given
Risk dialogue	DoR	Discussion of Risk
	RSRef	Risk score reference
	HARef	Heart age reference
	SARef	Survival age free of cardiac event reference
	RSM	Risk score manipulation using either QRISK2 or JBS3
	CVDRQ	Patient question about CVD risk
Causal CVD risk factors	FH-MHIS	Risk factor discussion – medical history (Med)
	RF-FH	Risk factor discussion – family history (Med)
	RF-W	Risk factor discussion – weight (Med)
	RF-C	Risk factor discussion – cholesterol (Med)
	RF-BP	Risk factor discussion – blood pressure (Med)
	RF-D	Risk factor discussion – diabetes (Med)
	RF-MH&W	Risk factor discussion – mental health & wellbeing (Med)
	RF-PA	Risk factor discussion – physical activity/inactivity (LS)
	RF-DIET	Risk factor discussion – diet (LS)
	RF-A	Risk factor discussion – alcohol consumption (LS)
	RF-S	Risk factor discussion – smoking (LS)
Risk management - Lifestyle interventions	I-W	Intervention discussion - weight
	I-C	Intervention discussion – cholesterol
	I-BP	Intervention discussion – blood pressure
	I-D	Intervention discussion – diabetes
	I-MW&W	Intervention discussion – mental health & wellbeing
	I-PA	Intervention discussion – physical activity
	I-DIET	Intervention discussion – diet
	I-A	Intervention discussion – alcohol
	IL-S	Intervention discussion – smoking
Risk management - Medical interventions	I-GP	Intervention discussion – GP appointment
	I-PR	Intervention discussion – practitioner appointment (practice nurse or HCA)
	I-M	Intervention discussion - medication
Comments & Recommendations		
