# Supplementary material for: Quantitative examination of video-recorded NHS Health Checks: comparison of the use of QRISK2 versus JBS3 cardiovascular risk calculators
Source: BMJ Open. 2020 Sep 25;10(9):e037790. doi: 10.1136/bmjopen-2020-037790 (PMC7520846; doi:10.1136/bmjopen-2020-037790)
Supplement: Author's manuscript [file bmjopen-2020-037790.draft_revisions.pdf]

BMJ Open is committed to open peer review. As part of this commitment we make the peer review history of every article we publish publicly available.

When an article is published we post the peer reviewers' comments and the authors' responses online. We also post the versions of the paper that were used during peer review. These are the versions that the peer review comments apply to.

The versions of the paper that follow are the versions that were submitted during the peer review process. They are not the versions of record or the final published versions. They should not be cited or distributed as the published version of this manuscript.

BMJ Open is an open access journal and the full, final, typeset and author-corrected version of record of the manuscript is available on our site with no access controls, subscription charges or pay-per-view fees (http://bmjopen.bmj.com).

If you have any questions on BMJ Open's open peer review process please email info.bmjopen@bmj.com

BMJ Open

The nature and content of NHS Health Checks: A quantitative comparison of video-recorded consultations using QRISK2 versus JBS3 CVD risk calculators

Journal:	*BMJ Open*
Manuscript ID	bmjopen-2020-037790
Article Type:	Original research
Date Submitted by the Author:	17-Feb-2020
Complete List of Authors:	Gidlow, Christopher; Staffordshire University, Centre for Health and Development<br>Ellis, N; Staffordshire University, Centre for Health and Development<br>Cowap, Lisa; Staffordshire University, Department of Psychology<br>Riley, V; Staffordshire University, Centre for Health and Development<br>Crone, Diane; Cardiff Metropolitan University<br>Cottrell, Elizabeth ; Keele University, School of Primary, Community and Social Care<br>Grogan, S; Manchester Metropolitan University,<br>Chambers, Ruth; NHS Stoke on Trent,<br>Clark-Carter, David; Staffordshire University, Department of Psychology
Keywords:	PREVENTIVE MEDICINE, PRIMARY CARE, Coronary heart disease < CARDIOLOGY

SCHOLARONE™
Manuscripts

I, the Submitting Author has the right to grant and does grant on behalf of all authors of the Work (as defined in the below author licence), an exclusive licence and/or a non-exclusive licence for contributions from authors who are: i) UK Crown employees; ii) where BMJ has agreed a CC-BY licence shall apply, and/or iii) in accordance with the terms applicable for US Federal Government officers or employees acting as part of their official duties; on a worldwide, perpetual, irrevocable, royalty-free basis to BMJ Publishing Group Ltd ("BMJ") its licensees and where the relevant Journal is co-owned by BMJ to the co-owners of the Journal, to publish the Work in this journal and any other BMJ products and to exploit all rights, as set out in our licence.

The Submitting Author accepts and understands that any supply made under these terms is made by BMJ to the Submitting Author unless you are acting as an employee on behalf of your employer or a postgraduate student of an affiliated institution which is paying any applicable article publishing charge ("APC") for Open Access articles. Where the Submitting Author wishes to make the Work available on an Open Access basis (and intends to pay the relevant APC), the terms of reuse of such Open Access shall be governed by a Creative Commons licence – details of these licences and which Creative Commons licence will apply to this Work are set out in our licence referred to above.

Other than as permitted in any relevant BMJ Author's Self Archiving Policies, I confirm this Work has not been accepted for publication elsewhere, is not being considered for publication elsewhere and does not duplicate material already published. I confirm all authors consent to publication of this Work and authorise the granting of this licence.

For peer review only

Title

The nature and content of NHS Health Checks: A quantitative comparison of video-recorded consultations using QRISK2 versus JBS3 CVD risk calculators

Authors

Christopher J. Gidlow, Staffordshire University, Brindley Building, Leek Road, Stoke-on-Trent, ST4 2DF, c.gidlow@staffs.ac.uk

Naomi J. Ellis, Staffordshire University, Brindley Building, Leek Road, Stoke-on-Trent, ST4 2DF, n.j.ellis@staffs.ac.uk

Lisa Cowap, Staffordshire University, Brindley Building, Leek Road, Stoke-on-Trent, ST4 2DF, l.cowap@staffs.ac.uk

Victoria Riley, Staffordshire University, Brindley Building, Leek Road, Stoke-on-Trent, ST4 2DF, victoria.riley@staffs.ac.uk

Diane Crone, Cardiff Metropolitan University, Cyncoed Campus, Cyncoed Road, Cardiff, CF23 6XD, dmcrone@cardiffmet.ac.uk

Elizabeth Cottrell, Keele University, Keele, Newcastle-under-Lyme, ST5 5BG, e.cottrell@keele.ac.uk

Sarah Grogan, Manchester Metropolitan University, Manchester Campus, Bonsall Street, Manchester, M15 6GX, s.grogan@mmu.ac.uk

Ruth Chambers, Stoke-on-Trent Clinical Commissioning Group, Smithfield One Building, Stoke-on-Trent ST1 4FA, Ruth.Chambers@stoke.nhs.uk

David Clark-Carter, Staffordshire University, The Science Centre, Leek Road, Stoke-on-Trent, ST4 2DF, d.clark-carter@staffs.ac.uk

*Corresponding author

For peer review only

Abstract

Objectives

Compare the nature and content of NHS Health Check (NHSHC) consultations, particularly the extent of cardiovascular disease (CVD) risk communication, when using QRISK2 or JBS3 CVD risk calculators.

Design

RIsk COmmunication in NHS Health Check (RICO) is a qualitative study with quantitative process evaluation, comparing NHSHC delivered using QRISK2 or JBS3. We present data from the quantitative process evaluation.

Setting and participants

Twelve general practices (6/group) were recruited in the West Midlands of England. Patients were those eligible for NHSHC based on national criteria (40-74 years old, no existing diagnoses for cardiovascular-related conditions, not on statins). Recruitment was stratified by patient age, gender and ethnicity.

Methods

NHSHC were video-recorded. Recordings were coded second-by-second to quantify who was speaking and what was being discussed. Outcomes included consultation duration, practitioner verbal dominance (ratio of practitioner:patient speaking time), and proportion of time discussing CVD risk, risk factors and risk management.

Results

A total of 173 video-recorded NHSHC were analysed (73 QRISK, 100 JBS3). The sample was 51% female, 83% White British ethnicity, with approximately equal proportions across age groups. NHSHC duration varied (6.8 to 38 minutes). Compared with JBS3, NHSHC using QRISK2 were slightly shorter (QRISK2 19.2±6.8 vs. JBS3 20.7±5.7%, p=.02) and more verbally dominated by practitioners (practitioner:patient ratio 3.2±1.6 vs. 2.4±1.3, p<.001). Overall, 9.1±4.3% of total consultation time was spent discussing CVD risk (1.7±.8 minutes), but this was higher when using JBS3 (QRISK2 7.4±4.1 vs. JBS3 10.2±4.1%, p<.001). The largest proportion of NHSHC time was spent discussing causal risk factors, with a small-medium between-group difference (QRISK2 40.6±11.9 vs. JBS3 35.3.6±13.3%, p<.001).

Conclusions

There was wide variation in NHSHC duration. Many were short and practitioner-dominated, with limited time discussing CVD risk. Our data suggest that JBS3 could provide a useful tool to improve delivery and strengthen the case for practitioner training.

Trial registration: ISRCTN ISRCTN10443908. Registered 7th February 2017.

Keywords: Cardiovascular disease; risk communication; Health Check; chronic disease prevention

For peer review only

Strengths and limitations of this study

- Video-recording of NHS Health Checks provide an objective record of practice across a range of general practices.
- Second-by-second coding was used to characterise the content of Health Checks that used either QRISK2 or JBS3 CVD risk.
- The study was limited to 12 general practices in the West Midlands of England
- Target sample size was not achieved.
- Qualitative data are necessary to provide further insight.

For peer review only

Introduction

Cardiovascular disease (CVD) accounts for over a quarter of UK deaths and costs the National Health Service (NHS) around £7 billion annually (1). Despite decreasing CVD mortality [1], prevention remains a priority and the NHS Health Check (NHSHC) programme remains an important part of this [2,3]. NHSHC aims to assess CVD risk of adults in England aged 40-74 years who are not known to have certain cardiovascular-related diseases [4]. It is the largest CVD risk identification and management programme of its kind globally, and has been linked with some increases in the detection of risk factors and chronic disease, and statin prescriptions [5,6]. NHSHC consultations typically take place in primary care with a primary care nurse, and comprise: (i) assessing patients' CVD risk, (ii) communication of CVD risk, which should inform (iii) discussion of CVD risk management through lifestyle or subsequent medical appointments or referrals.

To date, little is known about the nature or content of NHSHC consultations. There are gaps in the evidence, relating to long-term benefits of NHSHC for CVD risk, morbidity and mortality [7–12], with mixed predictions of future benefit from microsimulation studies [13,14]. The little we know about what takes place in the consultations is mainly limited to qualitative data from retrospective interviews with patients and practitioners asked to recall and reflect on their experiences [15]. Whilst these data have value, they do not present a complete understanding of the dynamics and interactions that may influence the long-term outcomes of an NHSHC. Patient outcomes for NHSHC will depend on their actions, and the support and interventions available to them following their consultation (e.g., referral of patients to effectiveness lifestyle support programmes, appropriate specialist referrals). There is evidence that patient outcomes from primary care consultations are influenced by their experience, hence the need to understand more about the dynamics of the NHSHC event. If delivery of the NHSHC consultation does not create a positive experience for patients, their engagement with, and effectiveness of subsequent risk management actions, could be undermined. To optimise their efficacy as a lifestyle behaviour intervention, a better understanding of what is already occurring within NHSHC consultations and identification of areas requiring improvement is necessary.

For CVD risk assessment in NHSHC, QRISK® (QRISK2 and, more recently, QRISK3) is the mandated CVD risk calculator [16,17]. QRISK provides a percentage risk of a CVD event in the next 10 years; this has to be communicated to patients for the NHSHC to be considered 'complete'. It is integrated within the general practice electronic medical record software, so can be calculated from pre-populated and new data. The score is then directly saved into the patient's record. However, there are limitations to the QRISK score and how it is used. First, 10-year risk estimates, such as those presented by QRISK have been criticised for being heavily influenced by age and gender, thereby underestimating risk in younger adults and women, and not accounting for risk from other diseases as effectively as long-term (lifetime) estimates [18,19]. Second, qualitative studies indicate limited practitioner and patient understanding of percentage CVD risk [15]. Practitioners report difficulties in explaining percentage CVD risk [20–23]. In turn, patients attending NHSHCs have been unable to recall being provided with a risk score or find it confusing [15].

In 2014, Joint British Societies recommendations on the prevention of Cardiovascular Disease (JBS3) launched the JBS3 risk calculator. JBS3 has a primary focus on lifetime risk [18], but includes heart age [7,24–26] and uses multiple visual displays to present them (e.g., Cates plot, image of heart for heart age, visual analogue scales) [27]. It has also been designed to allow manipulation to show the effects of risk factor modification (e.g., smoking cessation) on lifetime risk trajectory. There is some evidence that lifetime risk can identify raised CVD risk in some people that would not be picked up through conventional 10-year risk estimates [28]; that heart age is more easily communicated to, and recalled

For peer review only - http://bmjopen.bmj.com/site/about/guidelines.xhtml

by, patients [29,30]; and that graphical displays can be preferable for promoting risk-reducing behaviour [31]. Collectively, these attributes of JBS3 might accommodate a range of patients and facilitate practitioner communication [24,27], but a comparison of the relative benefits JBS3 and QRISK2 for communicating CVD risk in NHSHC has not yet been undertaken. Knowing which approach best delivers the information that patients need to support behaviour change will enable practices to make informed decisions about practitioner training and resource allocation.

Another potentially important, but so far unexplored feature of interactions within NHSHC consultations, is the practitioner-patient balance, particularly the extent to which patients are engaged in discussion of their CVD risk and how to manage it. Ideally, CVD risk management should be negotiated through a mutual exchange between practitioner and patient as part of a shared decision making process [32]. The NHSHC practitioner competencies include the need for a person-centred approach, which places the individual at the centre of their own care, service or treatment. Studies of clinician-patient interactions have identified that short, clinician-dominated (or 'paternalistic') consultations, are less patient-centred and linked with low patient and clinician satisfaction [33–36], which in turn, have been linked with poorer patient outcomes, such as adherence to clinical recommendations and health-promoting behaviour [37].

In summary, NHSHC aims to assess CVD risk and prompt patients and practitioners to undertake risk management behaviours. To date, there is insufficient knowledge about how they are conducted, the nature and adequacy of CVD risk communication, and the potential benefit of using alternative CVD risk calculators, like JBS3. The RIsk COmmunication in NHS Health Check (RICO) is a large study of practitioner and patient perceptions and understanding of CVD risk when using the JBS3 or QRISK®2 CVD risk calculators. Video-recording methods in RICO have provided the first objective data of NHSHC in practice, allowing extensive qualitative and quantitative analysis of consultations. We present the first data from quantitative coding of video-recorded NHSHC within RICO, with three aims: (i) quantitatively describe the content of NHSHC consultations, particularly the time devoted to CVD risk discussion; (ii) explore the level of practitioner verbal dominance; (iii) compare i and ii when NHSHC are conducted using QRISK2 versus JBS3 CVD risk calculators.

Method

Study design

The RICO study is a qualitative study with quantitative process evaluation. A detailed description of the overall study, including sampling, recruitment and data collection is available [38]. Here, we report a quantitative comparison of the content of video-recorded NHSHC consultations.

The RICO protocol was approved the Health Authority Approval (11/11/2017) and the London-Dulwich Research Ethics Committee (11/09/17, ref: 17/LO/1463). Written informed consent was obtained for all participants prior to taking part.

Public and Public Involvement and Engagement

Our approach was informed by extensive Patient and Public Involvement and Engagement (PPIE). Engagement with Patient Participation Groups at three general practices on two occasions was used to gather opinion on the study concept and overall design, and subsequently, the methods and protocols. Four mock NHSHC were used to test protocols, such as camera placement, video-recording

For peer review only - http://bmjopen.bmj.com/site/about/guidelines.xhtml

quality, participant consent and debrief processes and for development of the coding framework. Two patient representatives sit on the Study Steering Committee for ongoing involvement of patients in project management and a virtual study patient group was established using a closed Facebook group (>260 members) for ongoing PPIE.

Setting and general practice recruitment

Data were collected across 12 general practices in the West Midlands of England that already delivered NHSHC (January 2017 to February 2019). Practices were recruited via the local Clinical Research Network (CRN). Six practice pairs, approximately matched on deprivation, were randomly assigned to one of two groups: QRISK®2 (usual practice) - practitioners continue to use QRISK®2 to communicate CVD risk; JBS3 (intervention) - practitioners use the JBS3 CVD risk calculator following brief training about the platform (no training regarding risk communication was provided). As a minimum, those using JBS3 were asked to use the first two output screens from JBS3 showing: (i) Heart Age - estimate of heart age compared with someone of the same gender, ethnicity and risk factors at optimal levels; (ii) Survival age – estimate of the age the patient can expect to reach without a CVD event. They were also asked to show patients the effect of intervention on one or more of the CVD risk metrics (e.g., effect of smoking cessation on survival age). A brief training video was created to guide clinicians (https://www.youtube.com/watch?v=idecGzlwIc4&feature=youtu.be).

Participants and recruitment

Patients were those eligible for NHSHC based on national criteria. Thus people were excluded if they were outside of the target age range (40-74 years), had existing diagnoses for certain cardiovascular-related chronic conditions, took statins, had received an NHSHC in the last five years, or were known to be at high risk of CVD [39]. Within each practice, the list of eligible patients was stratified according to age, gender and ethnicity, so that invitations were sent to a representative sample. Postal invitations were distributed and follow-up calls were made by practice staff.

Practitioners all worked within primary care (8 Health Care Assistants, 6 Practice Nurses, 1 Sister) and already undertook NHSHC as part of routine practice. The only exception was one HCA who was new to NHSHC delivery.

Data sources and processing

Participating practices were asked to video-record NHSHCs using the allocated CVD risk calculator until 20 useable consultations were recorded (with written permission from patients and practice consent). Video-recorded NHSHC consultations were the main data source. Recorded NHSHC were viewed by Authors LC and VR and the content of consultations characterised using a second-by-second coding framework developed specifically for this study. The framework comprised 36 items grouped in to six categories: patient-practitioner communication, Health Check general (e.g., collecting and inputting data), risk dialogue, CVD risk factors, lifestyle interventions, medical interventions (Supplementary file, Table S1). This allowed derivation of aggregate indicators for each consultation, to allow between-group comparisons:

- Length of NHSHC

For peer review only - http://bmjopen.bmj.com/site/about/guidelines.xhtml

- Time and proportion of consultation time discussing CVD risk, CVD risk factors (overall, lifestyle, medical), and risk management (lifestyle, medical)
- Practitioner verbal dominance (ratio of practitioner: patient speaking time)
- Number and proportion of patients asking questions about CVD risk
- Use of Heart Age, Survival age and risk score manipulation (as fidelity check in the JBS3 group).

As noted previously [38], the coding process and framework development was iterative, using four mock NHSHC that were video-recorded as part of PPIE. To reach consistency in approach, Authors NE and LC coded mock NHSHC by consensus. Author VR then coded the same four consultations independently and Intraclass Correlation Coefficients (ICCs) demonstrated excellent inter-rater reliability (ICCs from .968 to .995). Once data collection had begun, NHSHC were coded by Authors LC and VR, with verification of 10% (two in every 20 independently coded) to mitigate the risk of coder drift throughout the study.ICCs ranging from .992 to .999 indicated excellent inter-rater reliability.

Data were also collected on patient sex, age and ethnic background [classified as White British (WBRI) or Black Minority Ethnic (BAME)].

Sample size

The target sample size was 240 (120 per group). A sample size calculation undertaken for the desired between-group quantitative comparison estimated that 120 consultations per group, using a between subjects t-test with a two-tailed probability and alpha of .05, would provide statistical power of at least .8 to detect an effect size (r) = 0.18 (small to medium effect).

Statistical methods

Following checks for normal distributions, QRISK2 and JBS3 groups were compared on key variables. Test for skewness and kurtosis, and visual inspections (histograms, Q-Q plots) were used to check for normal distribution. All but one of the continuous variables (% of NHSHS time discussing CVD risk) were positively skewed and, therefore, square root transformed. Two-tailed between-subjects t-tests were used to assess between-group differences in untransformed and square root-transformed continuous variables. The Mann-Whitney U-test was used where transformations were not possible (% NHSHC time discussing medical intervention) and chi-squared tests used for categorical binary variables (use of Heart Age, survival age and risk score manipulation, and whether patients asked questions about CVD risk). Data processing and analysis were performed in SPSS 26 and Stata 14.

Results

Participants

A total of 175 video-recorded NHSHC were completed, of which 173 were included in analysis (QRISK=73; JBS3=100; 2 excluded for practitioner error in NHSHC that invalidated the consultation). The sample comprised approximately equivalent proportions of males and females, and proportions in White British versus BAME ethnic groups that were representative of the geographical region (Table 1). Age of the JBS3 group was higher on average than QRISK2 (Mdn=62 vs. 55, U=2312.5, p<.001), but CVD risk scores were not significantly different between the two groups (mean difference =1.03, 95%CI -1.15, 3.20, p=.098).

For peer review only - http://bmjopen.bmj.com/site/about/guidelines.xhtml

Length of NHS Health Check consultations

Table 2 summarises the characteristics of NHSHC consultations by group, and overall. There was a wide range in consultation length, ranging from just 6.8 to over 38 minutes, but the majority were between 15 and 20 minutes (Figure 1). Consultations were significantly shorter in the QRISK2 compared with JBS3 group (with a relatively small effect size of .13).

Discussion of CVD risk

Overall, less than 10% of overall consultations time was devoted to CVD risk discussion, which equated to 1.7±0.83 minutes. A higher proportion of consultation time was spent discussing CVD risk using JBS3 (2.1±0.82 minutes) compared with QRISK2 (1.31±0.63 minutes), with a medium effect size. Nearly all NHSHC in both groups included reference to the 10-year % CVD risk score (94 vs. 94.5%, χ^2=.021, p=.885, r = .01). The proportion of patients asking questions about CVD risk was higher in the JBS3 versus QRISK2 group (32.0 vs. 12.3%, χ^2=9.03, p=.003, r=.23).

Within the JBS3 group, nearly all practitioners discussed Heart Age (100%) and Survival age (97%), and manipulated risk score to show the potential effect of intervention on risk (92%). QRISK2 can also provide Heart Age and allow manipulation, which was evident in 52.1% and 21.9% of QRISK consultations, respectively.

Discussion of CVD risk factors and risk management

Over one-third of total NHSHC time was spent discussing CVD risk factors. This was higher in the QRISK2 versus JBS3 informed NHSHCs, overall and for medical and lifestyle-related risk (Table 2).

Interventions to manage risk were discussed for approximately one-fifth of total consultation time and predominantly related to lifestyle, rather than medical intervention. There were no significant between-group differences.

Verbal dominance

Practitioners spoke for just over half of total time in QRISK2 consultations and just under half in JBS3 (Figure 2). Verbal dominance ratio was higher in NHSHC using QRISK2 versus JBS3 consultations.

Discussion

Main findings

We present the first objective data on the nature and content of NHSHC consultations, with comparison of QRISK2 and JBS3 CVD risk calculators. Second-by-second coding of 173 video-recorded NHSHC consultations from 12 general practices provided new insights regarding duration, verbal dominance and allocation of time to different components of the consultation. Main findings in relation to our study aims were as follows. First, the length of NHSHC consultations was varied and often short (most <20 minutes). Less than 10% of time (<2 minutes) was spent discussing the

For peer review only - http://bmjopen.bmj.com/site/about/guidelines.xhtml

individual's calculated CVD risk, and the largest component was discussion of CVD risk factors (particularly medical). Second, there was evidence of practitioner verbal dominance as, on average, practitioners spoke for half of total consultation time (compared with ~23% with patients speaking). Third, compared with QRISK2, use of JBS3 was associated with more discussion of CVD risk, less discussion of CVD risk factors, reduced practitioner verbal dominance and, on average, required an additional 1.4 minutes (<90 seconds).

What this study adds

The variable and often short duration of NHSHC, combined with practitioner verbal dominance are a potential concern. Sixty per cent of NHSHC in the RICO study lasted less than 20 minutes, 18% less than 15 minutes, and 4% were under 10 minutes. Nearly all (95%) had a verbal dominance ratio greater than one, 57% had a ratio of two or more, and 36% were over three, which indicates some degree of verbal dominance. Practitioner verbal dominance suggests more information provision than mutual exchange. While information provision is an important part of the process, the literature on clinician-patient interactions suggests that short, paternalistic primary care consultations are associated with low patient and clinician satisfaction [36]. This can have negative implications for patient outcomes, potentially reducing adherence to clinician recommendations, ability to self-care or make informed decision about their health [33–35].

Practitioner-patient interactions are complex [40]. The practitioner-patient balance has been discussed as a spectrum of locus of control, with a paternalistic health professional at one end and the 'informed patient' at the other [41]. The latter requires that control is mutual or exchanged, allowing a 'negotiated plan'; in this case, a negotiated plan to manage CVD risk. The nature of interactions have been conceptualised and measured variously. For example, 'patient enablement', whereby patients have understanding, confidence and coping ability following enabling consultations [33], and 'physician–patient collaboration', where both physician and patient are active participants in consultations [42]. A common feature of consultations that foster these beneficial relationships (and should lead to better outcomes) is the active participation of patients in the consultation and decisions about their health care. Our data indicate that there is scope in NHSHC for greater patient participation, especially as motivational interviewing should be a key feature of NHSHC and relies on a patient-centred approach [16].

The length of appointments that practices gave to NHSHC ranged from 15 to 30 minutes. This showed variation in the time that general practices were willing to give to NHSHC. Yet, video-recordings highlighted even greater variability in the actual duration of practitioner-patient interaction. The length of primary care nurse appointments varies by appointment type. NHSHC require that practitioners undertake CVD risk assessment (sometimes involving point-of-care testing, data entry), explain that risk and what it means, which should inform a patient-centred discussion of risk management. Our data highlight a need to consider appropriate appointment length to ensure that practitioners have sufficient time to deliver all parts of the NHSHC, with appropriate levels of patient participation as part of a mutual exchange.

A primary focus of the RICO project is CVD risk communication in NHSHC. To promote health-protective behaviours that reduce CVD risk, practitioners need to understand the risk information and be able to communicate it effectively such that patients leave the consultation with the knowledge and intention to act [31]. The little time dedicated to CVD risk discussion (<10%), especially in when using QRISK2, accords with findings from the 2017 systematic review of qualitative data studies [43].

For peer review only - http://bmjopen.bmj.com/site/about/guidelines.xhtml

The review authors identified patients' 'limited understanding of the risk score' as a common theme. Some patients reported improved understanding of CVD risk following their NHSHC, but many did not recall being told their risk, found it confusing or had misinterpreted their risk score.

Our data confirmed that nearly all practitioners using JBS3 conveyed heart age, survival age and used risk score manipulation (as requested). The additional proportion of that time spent on CVD risk discussion in NBS3-informed consultations, was significant but equated to less than one minute on average. This raises the question of whether JBS3 does help to engender better patient engagement and understanding of their risk, or simply more information provision. That one in three patients in JBS3 consultations asked questions about their CVD risk (32%), compared with one in eight QRISK2 patients (12%), provides evidence of better engagement, but qualitative analysis is required to provide a more complete understanding. RICO did not include extensive practitioner training in use of JBS3. Rather, an introductory briefing with supporting DVD were used to maintain the ecological validity of exploring how practitioners would use JBS3 if made available in practice (not the effect of additional training, which is generally not available in practice) [44]. Such training might be required and general CVD risk communication training for NHSHC practitioners has shown some benefits for practitioner confidence and understanding [44].

The apparent focus on causal risk factors, discussion of which comprised 38% of NHSHC time overall (over 40% when using QRISK2), is perhaps an indication that practitioners did not to dwell on risk score and what it means, but quickly move on to the potential causes and their management. This could be interpreted as a positive; a solution-focused approach that focuses on the tangible factors that might be easily understood; i.e., how their medical history and lifestyle can lead and help to prevent CVD. However, it is important that this discussion is in the context of the patient's CVD risk and tailored to their lifestyle and priorities. Risk communication is challenging [27]. If delivered effectively, it can enhance knowledge and decision making about treatment, and can empower and create autonomy [45]. Conversely, poor communication of risk can cause patients anxiety and reduce confidence in health professionals, or may result in the perception that action is futile [46]. Therefore, the 38% and 20% of NHSHC time spent discussing CVD risk factors and management could be undermined if the patient is confused or alarmed by the preceding information about their CVD risk.

Implications for practice

The 2019 green paper, *Advancing our health: prevention in the 2020s*, set out plans for an evidence-based review of NHSHC to maximise benefit in the next decade (18). This indicates a future for NHSHC, with evidence-based changes to delivery. Despite the important of risk communication, associated recommendations in NHSHC best practice guidance [16] and the practitioner competency framework that includes CVD risk communication [47], we have provided the first objective data showing the varied and often short NHSHC, the limited time devoted to CVD risk discussion, and that this can be increased through using JBS3. We know that training in NHSHC is a challenge to optimal implementation [15], practitioners generally receive little (or no) training in CVD risk communication for NHSHC, and can lack the associated confidence and skills [44]. Our data strengthen the case for NHSHC practitioner training, ideally with patient involvement, to redress the practitioner verbal dominance and help to make the discussion of CVD risk appropriate and positive for patients. JBS3 (or similar tools) could form part of this solution.

For peer review only - http://bmjopen.bmj.com/site/about/guidelines.xhtml

Strengths and limitations

The strengths of this study include video-recording of NHSHC across a diverse range practices stratified by deprivation, stratified sampling of patients, and our comprehensive coding approach. A number of limitations are recognised. First, challenges with practice and patient recruitment meant that we did not hit our target sample size of 240 (120/group). However, power of greater than 0.8 would have been achieved with an effect size of r = .22; thus the analyses were adequately powered for results below a medium effect size. Second, there was a between-group difference in mean age. This did not translate into a difference in CVD risk, which would have introduced more systematic bias into the sample. But this would mean that risk was underestimated in larger proportion of younger people in the QRISK2 group (compared with the JBS3 group). Third, these quantitative data do not tell us about quality of discussion or patient response. Qualitative analysis is required to provide these insights. Third, we cannot claim that our results are generalizable to the rest of England. However, we have a good balance of males/females, a good age range and an ethnic mix that was representative of the region. Fourth, after commencing data collection, we discovered that one QRISK2 practice used Informatica, an addition to practice software that has some of the JBS3 functionalities. To maintain the ecological validity of a 'usual care' group, the 16 patients from this practice were included in main analysis. Sensitively analysis confirmed that their exclusion did not alter the overall effects described, except that the greater proportion of NHSHC time discussing lifestyle interventions in the QRISK2 group (compared with JBS3), was no longer significant (Supplementary file, Table S2). Finally, it is possible that being video-recorded affected practitioner behaviour (Hawthorne effect). To mitigate this, our PPIE explored camera position to best capture patient response and minimise practitioner awareness of the camera, and practitioners in mock NHSHC reported forgetting about the camera during consultations. The Hawthorne effect tends to improve performance [48], which would mean that any effect would lead to an underestimate of issues with NHSHC delivery.

Conclusions

Duration of the practitioner-patient interaction in NHSHC is varied and, in many cases, short. Our data suggest a need for greater time on CVD risk discussion, for which JBS3 could provide a useful tool. Practitioner verbal dominance should be further explored in relation to patient experience and outcomes. Further qualitative data and analysis will help to provide a more complete picture and elucidate the true potential of JBS3, but data presented here strengthen the case for practitioner training. Public Health England's competency framework for NHSHC specifies that NHSHC practitioners '*should be trained in communicating the risk score and results to the client*' and that '*methods, such as motivational interviewing techniques, should engage clients in person-centred conversations about their own reasons for change*' [47, p21]. The need for professional development in this area is building, and could involve additions to standard competencies within health professional training.

For peer review only - http://bmjopen.bmj.com/site/about/guidelines.xhtml

Funding

This project is funded by the National Institute for Health Research HTA Programme (project number 15/170/02). The funders had no role in study design, data collection and analysis, decision to publish, or preparation of the manuscript.

Ethics approval and consent to participate

The study was approved by the Health Authority Approval (11/11/2017) and the London - Dulwich Research Ethics Committee (11/09/17) (reference: 17/LO/1463). Written informed consent will be obtained for all participants prior to taking part.

Competing interests

The authors declare that they have no competing interests.

Authors' contributions

CG, DC, NE, EC, SG and RC contributed to the conceptualisation and design. NE, LC and CG led the development of the coding system and DCC advised on verification testing. LC and VR completed coding of recorded NHSHC. NE, LC and VR contributed to the Patient Public Involvement. CG completed statistical analysis (supported by DCC) and manuscript preparation. All authors contributed to the writing of the manuscript. All authors read and approved the final manuscript.

Acknowledgements

The authors would like to acknowledge Clinical Research Network West Midlands colleagues for advice in developing protocols, and members of the PPG and the virtual PPI participants who have informed study development.

Data availability

No additional data are available.

For peer review only

References

1 Bhatnagar P, Wickramasinghe K, Wilkins E, et al. Trends in the epidemiology of cardiovascular disease in the UK. Heart 2016;102:1945–52.

2 National Health Service. The NHS Long Term Plan. London: NHS 2019.

3 Department of Health and Social Care. Advancing our health: prevention in the 2020s – consultation document. London: HMSO 2019.

4 Department of Health. Putting prevention first. NHS Health Check: vascular risk assessment and management best practice guidance. London: Department of Health 2009.

5 Robson J, Dostal I, Sheikh A. The NHS health check in England: an evaluation of the first 4 years. BMJ Open 2016;6.

6 Forster AS, Burgess C, Dodhia H, et al. Do health checks improve risk factor detection in primary care? Matched cohort study using electronic health records. J Public Health 2016;38:552–9.

7 Soureti A, Hurling R, Murray P, et al. Evaluation of a cardiovascular disease risk assessment tool for the promotion of healthier lifestyles. Eur J Cardiovasc Prev Rehabil 2010;17:519–23.

8 Gøtzsche PC, Jørgensen KJ, Krogsbøll LT. General health checks don't work. Br Med J 2014;348.

9 Caley M, Chohan P, Hooper J, et al. The impact of NHS Health Checks on the prevalence of disease in general practices: a controlled study. Br J Gen Pract 2014;64:e516–21.

10 Si S, Moss JR, Sullivan TR, et al. Effectiveness of general practice-based health checks: a systematic review and meta-analysis. Br J Gen Pract 2014;64:e47–53.

11 Chang KC-M, Lee JT, Vamos EP, et al. Impact of the National Health Service Health Check on cardiovascular disease risk: a difference-in-differences matching analysis. Can Med Assoc J 2016;188(10):E228-E23.

12 Krogsbøll LT, Jørgensen KJ, Larsen CG, et al. General health checks in adults for reducing morbidity and mortality from disease: Cochrane systematic review and meta-analysis. BMJ 2012;345. doi:10.1136/bmj.e7191

13 Mytton OT, Jackson C, Steinacher A, et al. The current and potential health benefits of the National Health Service Health Check cardiovascular disease prevention programme in England: A microsimulation study. PLoS Med 2018;15:e1002517.

14 Kypridemos C, Allen K, Hickey GL, et al. Cardiovascular screening to reduce the burden from cardiovascular disease: microsimulation study to quantify policy options. BMJ 2016;353:i2793.

15 Usher-Smith JA, Mant J, Martin A, et al. NHS Health Check Programme Rapid Evidence Synthesis. Cambridge: The Primary Care Unit, University of Cambridge; RAND Europe 2017.

16 Public Health England. NHS Health Check Best practice guidance. London: Public Health England 2017.

17 National Institutes of Health and Care Excellence. Cardiovascular disease: risk assessment and reduction, including lipid modification. Clinical guideline [CG181]. London: NICE 2015.

18 JBS3 Board. Joint British Societies' consensus recommendations for the prevention of cardiovascular disease (JBS3). Heart 2014;100:ii1–67.

19 Karmali KN, Lloyd-Jones DM. Adding a life-course perspective to cardiovascular-risk communication. Nat Rev Cardiol 2013;10:111–5.

For peer review only

20 Gigerenzer G, Hoffrage U. How to improve Bayesian reasoning without instruction: Frequency formats. Psychol Rev 1995;102:684–704.

21 Hoffrage U, Gigerenzer G. Using natural frequencies to improve diagnostic inferences. Acad Med 1998;73:538–40.

22 Gigerenzer G, Edwards A. Simple tools for understanding risks: from innumeracy to insight. BMJ Br Med J 2003;327:741–4.

23 Slovic P, Monahan J, MacGregor DG. Violence risk assessment and risk communication: The effects of using actual cases, providing instruction, and employing probability versus frequency formats. Law Hum Behav 2000;24:271–96.

24 Wells S, Kerr A, Broadbent E, et al. Does Your Heart Forecast help practitioner understanding and confidence with cardiovascular disease risk communication? J Prim Heal Care 2011;3:4–9.

25 Bonner C, Jansen J, Newell BR, et al. I Don't Believe It, But I'd Better Do Something About It: Patient Experiences of Online Heart Age Risk Calculators. J Med Internet Res 2014;16.

26 Reed JL, Prince SA, Cole CA, et al. Workplace physical activity interventions and moderate-to-vigorous intensity physical activity levels among working-age women: a systematic review protocol. Syst Rev 2014;3:147.

27 Spiegelhalter D, Pearson M, Short I. Visualizing uncertainty about the future. Science (80-) 2011;333:1393–400.

28 Johns I, Moschonas KE, Medina J, et al. Risk classification in primary prevention of CVD according to QRISK2 and 'heart age', and prevalence of elevated high-sensitivity C reactive protein in the UK cohort of the EURIKA study. Open Hear 2018;5.

29 Hawking MKD, Timmis A, Wilkins F, et al. Improving cardiovascular disease risk communication in NHS Health Checks: a qualitative study. BMJ Open 2019;9.

30 Bonner C, Bell K, Jansen J, et al. Should heart age calculators be used alongside absolute cardiovascular disease risk assessment? BMC Cardiovasc Disord 2018;18:19.

31 Waldron C-A, van der Weijden T, Ludt S, et al. What are effective strategies to communicate cardiovascular risk information to patients? A systematic review. Patient Educ Couns 2011;82:169–81.

32 National Health Service. Shared decision making. 2019. Available at: https://www.england.nhs.uk/shared-decision-making (accessed 20 Nov 2019).

33 Pawlikowska T, Zhang W, Griffiths F, et al. Verbal and non-verbal behavior of doctors and patients in primary care consultations - How this relates to patient enablement. Patient Educ Couns 2012;86:70–6.

34 G Howie J, Heaney D, Maxwell M. Measuring quality in general practice. Pilot study of a needs, process and outcome measure. Occas Pap R Coll Gen Pract 1997;75:i–xii, 1.

35 Street RL, Makoul G, Arora NK, et al. How does communication heal? Pathways linking clinician–patient communication to health outcomes. Patient Educ Couns 2009;74:295–301.

36 Roter D, Stewart M, Putnam S, et al. Communication patterns of primary care physicians. JAMA 1997;277:350–6.

37 Doyle C, Lennox L, Bell D. A systematic review of evidence on the links between patient experience and clinical safety and effectiveness. BMJ Open 2013;3.

For peer review only - http://bmjopen.bmj.com/site/about/guidelines.xhtml

38 Gidlow CJ, Ellis NJ, Cowap L, et al. A qualitative study of cardiovascular disease risk communication in NHS Health Check using different risk calculators: protocol for the RIsk COmmunication in NHS Health Check (RICO) study. BMC Fam Pract 2019;20:11.

39 Public Health England. NHS Health Check Programme: Best practice guidance. London: PHE 2013.

40 Asan O, Montague E. Using video-based observation research methods in primary care health encounters to evaluate complex interactions. Inform Prim Care 2014;21:161—170.

41 Taylor K. Paternalism, participation and partnership—The evolution of patient centeredness in the consultation. Patient Educ Couns 2009;74:150–5.

42 Arbuthnott A, Sharpe D. The effect of physician–patient collaboration on patient adherence in non-psychiatric medicine. Patient Educ Couns 2009;77:60–7.

43 Usher-Smith JA, Harte E, Maclure C, et al. Patient experience of NHS health checks: A systematic review and qualitative synthesis. BMJ Open 2017;7.

44 Riley VA, Gidlow CJ, Ellis NJ, et al. Improving Cardiovascular Disease Risk Communication in the UK National Health Service Health Check Programme. Patient Educ Couns 2019;102:2016–23.

45 Ahl AS, Acree JA, Gipson PS, et al. Standardization of nomenclature for animal health risk analysis. Rev Sci Tech 1993;12:1045–53.

46 Thomson R, Edwards A, Grey J. Risk communication in the clinical consultation. Clin. Med. J. R. Coll. Physicians London. 2005;5:465–9.

47 Public Health England. NHS Health Check competence framework. London: PHE 2015.

48 Yanes AF, McElroy LM, Abecassis ZA, et al. Observation for assessment of clinician performance: a narrative review. BMJ Qual 2015; 25(1):46-55.

For peer review only

Figure titles:

Figure 1. Duration of NHSHC consultation by CVD risk calculator group

Figure 2. Mean percentage of total NHSHC time with speaking by practitioner, patient or no speaking, by CVD risk calculator group

Tables

Table 1. Participant characteristics

		JBS3		QRISK		Total		Difference		
		n	%	n	%	n	%	χ^2	p	r
Age	40-54	24	24.00	36	49.32	60	34.68	17.730	<.001	.32
	55-64	30	30.00	24	32.88	54	31.21			
	65-74	46	46.00	13	17.81	59	34.10			
	Total	100		73		173				
Gender	Male	49	49.00	37	50.68	86	49.71	0.480	0.474	.05
	Female	51	51.00	36	49.32	87	50.29			
	Total	100		73		173				
Ethnicity	WBRI	81	81.00	63	86.30	144	83.24	0.850	0.357	.07
	BAME	19	19.00	10	13.70	29	16.76			
	Total	100		73		173				
CVD risk cat	Low	57	57.00	47	64.38	104	60.12	0.697	0.41	.06
	Medium-High	41	41.00	26	35.62	67	38.73			
	Total	98		73		171				

Effect size, r: where .1 is small, .3 is medium, .5 is large

For peer review only

For peer review only - http://bmjopen.bmj.com/site/about/guidelines.xhtml

Table 2. Between-group comparisons of characteristics of NHSHC consultation

	JBS3		QRISK		Total		Difference (QRISK vs. JBS3)		
	Mean	SD	Mean	SD	Mean	SD	t	p	r
**Duration** (minutes)	20.66	5.65	19.24	6.84	20.06	6.21	-1.75	0.02	.13
**Verbal dominance**									
% of total time practitioner speaking	46.60	8.79	54.82	8.48	50.07	9.55	6.17	<.001	.42
% of total time patient speaking	24.67	10.53	21.6	10.56	23.37	10.62	-2.03	0.04	.15
Verbal dominance ratio [a]	2.35	1.31	3.18	1.62	2.70	1.50	3.72	<.001	.27
**% of total HC time:**									
Discussing CVD risk	10.24	4.07	7.44	4.08	9.06	4.30	-4.47	<.001	-.32
Discussing risk factors									
Total	35.33	13.29	40.58	11.91	37.54	12.96	2.97	<.001	.22
Medical [b]	20.13	9.38	22.98	9.26	21.34	9.41	2.20	0.03	.16
Lifestyle [c]	15.08	6.49	17.52	7.53	16.11	7.03	2.09	0.04	.16
Discussing risk management									
Total	18.82	11.12	20.77	11.69	19.64	11.37	1.09	0.28	.08
Lifestyle	15.94	10.04	17.48	10.41	16.59	10.2	1.01	0.32	.08
Medical [d]	2.88	3.37	3.29	4.01	3.05	3.65	3404 [d]	0.44	.06

Note: Analysis performed on square root-transformed variables, but untransformed descriptive data are presented
[a] Ratio of practitioner: patient talking (where higher value indicates greater practitioner verbal dominance);
[b] Includes medical history, family history, weight, blood pressure, cholesterol, diabetes, mental health and well-being;
[c] Includes diet, alcohol, physical activity, smoking
[d] Mann-Whitney U test used for between group comparison (test statistic reported is U)
Effect size, r: where .1 is small, .3 is medium, .5 is large

For peer review only - http://bmjopen.bmj.com/site/about/guidelines.xhtml

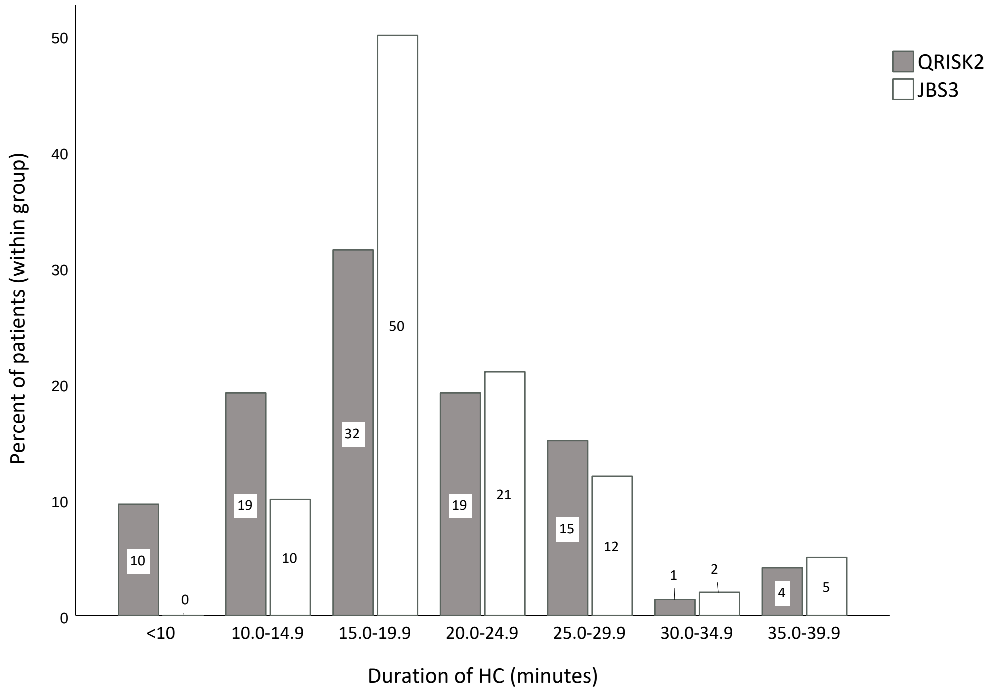

Page 1

For peer review only - http://bmjopen.bmj.com/site/about/guidelines.xhtml

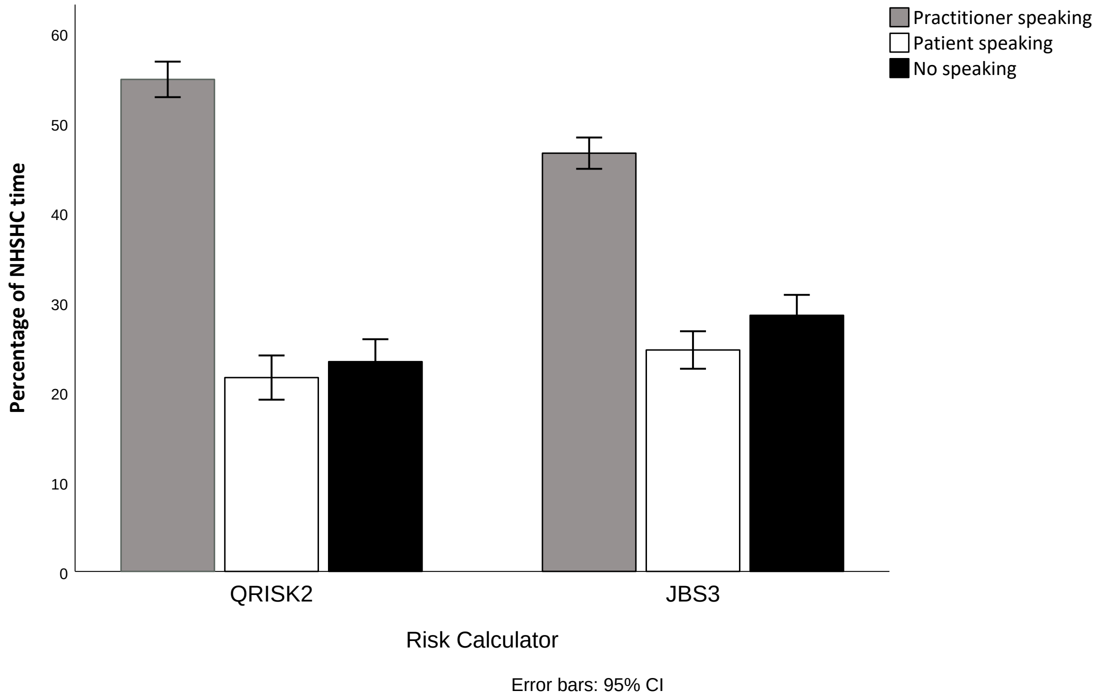

For peer review only - http://bmjopen.bmj.com/site/about/guidelines.xhtml

Table S1. Coding framework data variables

Category	Code	Code Title
Patient-practitioner communication	PrDT	Practitioner dominated talk
	PtDT	Patient dominated talk
	NT	No talking
Health Check general	I2HC	Introduction to Health Check
	HCSUM	Health Check summary
	C&ID	Collecting and Inputting Data
	TRG	Test result/recording given
Risk dialogue	DoR	Discussion of Risk
	RSRef	Risk score reference
	HARef	Heart age reference
	SARef	Survival age free of cardiac event reference
	RSM	Risk score manipulation using either QRISK2 or JBS3
	CVDRQ	Patient question about CVD risk
CVD risk factors	FH-MHIS	Risk factor discussion – medical history
	RF-FH	Risk factor discussion – family history
	RF-W	Risk factor discussion – weight
	RF-C	Risk factor discussion – cholesterol
	RF-BP	Risk factor discussion – blood pressure
	RF-D	Risk factor discussion – diabetes
	RF-MH&W	Risk factor discussion – mental health & wellbeing
	RF-PA	Risk factor discussion – physical activity/inactivity
	RF-DIET	Risk factor discussion - diet
	RF-A	Risk factor discussion – alcohol consumption
	RF-S	Risk factor discussion – smoking
Lifestyle interventions	I-W	Intervention discussion - weight
	I-C	Intervention discussion – cholesterol
	I-BP	Intervention discussion – blood pressure
	I-D	Intervention discussion – diabetes
	I-MW&W	Intervention discussion – mental health & wellbeing
	I-PA	Intervention discussion – physical activity
	I-DIET	Intervention discussion – diet
	I-A	Intervention discussion – alcohol
	IL-S	Intervention discussion – smoking
Medical interventions	I-GP	Intervention discussion – GP appointment
	I-PR	Intervention discussion – practitioner appointment (practice nurse or HCA)
	I-M	Intervention discussion - medication
Comments & Recommendations		

For peer review only

Table S2. Sensitivity analysis – analysis repeated with exclusion of patients from the general practice that used Informatica

	JBS3 (n=100		QRISK2 (n=151)		Total		Difference (QRISK vs. JBS3)	
	Mean	SD	Mean	SD	Mean	SD	t	p
**Duration (minutes)**	20.66	5.65	17.63	7.1	19.63	6.32	-3.33	0.001
**Verbal dominance**								
% of total time practitioner speaking	46.6	8.79	54.34	8.65	49.22	9.45	5.14	<.001
% of total time patient speaking	24.67	10.53	24.67	10.53	24.48	10.72	-0.398	0.691
Verbal dominance ratio	2.35	1.31	2.88	1.56	2.53	1.41	2.18	0.031
**% of total time:**								
Discussing CVD risk								
Total	10.24	4.07	8.46	4.29	9.64	4.22	-2.46	0.016
Discussing risk factors								
Total	35.33	13.29	42.04	11.41	37.59	13.04	3.26	0.001
Medical	20.13	9.38	24.49	8.92	21.61	9.43	2.93	0.004
Lifestyle	15.08	6.49	15.08	6.49	15.88	7.1	1.68	**0.094**
Discussing interventions								
Total	18.82	11.12	21.34	12.52	19.67	11.63	1.08	0.28
Lifestyle	15.94	10.04	17.7	11.34	16.54	10.5	0.82	0.41
Medical	2.88	3.37	3.64	4.56	3.13	3.82	2363 [d]	0.45

STROBE Statement—checklist of items that should be included in reports of observational studies

	Item No	Recommendation (page information is reported on)
**Title and abstract**	1	(*a*) Indicate the study's design with a commonly used term in the title or the abstract (p.2)
		(*b*) Provide in the abstract an informative and balanced summary of what was done and what was found (p.2)
**Introduction**		
Background/rationale	2	Explain the scientific background and rationale for the investigation being reported (p.4)
Objectives	3	State specific objectives, including any prespecified hypotheses (p.5)
**Methods**		
Study design	4	Present key elements of study design early in the paper (p.4)
Setting	5	Describe the setting, locations, and relevant dates, including periods of recruitment, exposure, follow-up, and data collection (p.6)
Participants	6	(*a*) *Cohort study*—Give the eligibility criteria, and the sources and methods of selection of participants. Describe methods of follow-up<br>*Case-control study*—Give the eligibility criteria, and the sources and methods of case ascertainment and control selection. Give the rationale for the choice of cases and controls<br>*Cross-sectional study*—Give the eligibility criteria, and the sources and methods of selection of participants (p.6)
		(*b*) *Cohort study*—For matched studies, give matching criteria and number of exposed and unexposed<br>*Case-control study*—For matched studies, give matching criteria and the number of controls per case
Variables	7	Clearly define all outcomes, exposures, predictors, potential confounders, and effect modifiers. Give diagnostic criteria, if applicable (p.6-7)
Data sources/ measurement	8*	For each variable of interest, give sources of data and details of methods of assessment (measurement). Describe comparability of assessment methods if there is more than one group (p.7)
Bias	9	Describe any efforts to address potential sources of bias (p.6, 10-11)
Study size	10	Explain how the study size was arrived at (p.7)
Quantitative variables	11	Explain how quantitative variables were handled in the analyses. If applicable, describe which groupings were chosen and why (p.6-7)
Statistical methods	12	(*a*) Describe all statistical methods, including those used to control for confounding (p.7)
		(*b*) Describe any methods used to examine subgroups and interactions (n/a)
		(*c*) Explain how missing data were addressed (n/a)
		(*d*) *Cohort study*—If applicable, explain how loss to follow-up was addressed<br>*Case-control study*—If applicable, explain how matching of cases and controls was addressed<br>*Cross-sectional study*—If applicable, describe analytical methods taking account of sampling strategy (n/a)
		(*e*) Describe any sensitivity analyses (p.11, Supplementary file, Table S2)

Continued on next page

For peer review only - http://bmjopen.bmj.com/site/about/guidelines.xhtml

**Results**		
Participants	13*	(a) Report numbers of individuals at each stage of study—eg numbers potentially eligible, examined for eligibility, confirmed eligible, included in the study, completing follow-up, and analysed (n/a)
		(b) Give reasons for non-participation at each stage
		(c) Consider use of a flow diagram
Descriptive data	14*	(a) Give characteristics of study participants (eg demographic, clinical, social) and information on exposures and potential confounders (Table 1)
		(b) Indicate number of participants with missing data for each variable of interest (n/a)
		(c) *Cohort study*—Summarise follow-up time (eg, average and total amount)
Outcome data	15*	*Cohort study*—Report numbers of outcome events or summary measures over time
		Case-control study—Report numbers in each exposure category, or summary measures of exposure (Table 1)
		Cross-sectional study—Report numbers of outcome events or summary measures
Main results	16	(*a*) Give unadjusted estimates and, if applicable, confounder-adjusted estimates and their precision (eg, 95% confidence interval). Make clear which confounders were adjusted for and why they were included (n/a)
		(*b*) Report category boundaries when continuous variables were categorized (Table 2)
		(*c*) If relevant, consider translating estimates of relative risk into absolute risk for a meaningful time period (n/a)
Other analyses	17	Report other analyses done—eg analyses of subgroups and interactions, and sensitivity analyses (n/a)
**Discussion**		
Key results	18	Summarise key results with reference to study objectives (p.8-9)
Limitations	19	Discuss limitations of the study, taking into account sources of potential bias or imprecision. Discuss both direction and magnitude of any potential bias (p.11)
Interpretation	20	Give a cautious overall interpretation of results considering objectives, limitations, multiplicity of analyses, results from similar studies, and other relevant evidence (p.9-11)
Generalisability	21	Discuss the generalisability (external validity) of the study results (p.11)
**Other information**		
Funding	22	Give the source of funding and the role of the funders for the present study and, if applicable, for the original study on which the present article is based (p.12)

*Give information separately for cases and controls in case-control studies and, if applicable, for exposed and unexposed groups in cohort and cross-sectional studies.

Note: An Explanation and Elaboration article discusses each checklist item and gives methodological background and published examples of transparent reporting. The STROBE checklist is best used in conjunction with this article (freely available on the Web sites of PLoS Medicine at http://www.plosmedicine.org/, Annals of Internal Medicine at http://www.annals.org/, and Epidemiology at http://www.epidem.com/). Information on the STROBE Initiative is available at www.strobe-statement.org.

For peer review only - http://bmjopen.bmj.com/site/about/guidelines.xhtml

BMJ Open

Quantitative examination of video-recorded NHS Health Checks: comparison of use of QRISK®2 versus JBS3 cardiovascular risk calculators

Journal:	*BMJ Open*
Manuscript ID	bmjopen-2020-037790.R1
Article Type:	Original research
Date Submitted by the Author:	19-Jun-2020
Complete List of Authors:	Gidlow, Christopher; Staffordshire University, Centre for Health and Development<br>Ellis, N; Staffordshire University, Centre for Health and Development<br>Cowap, Lisa; Staffordshire University, Department of Psychology<br>Riley, V; Staffordshire University, Centre for Health and Development<br>Crone, Diane; Cardiff Metropolitan University<br>Cottrell, Elizabeth ; Keele University, School of Primary, Community and Social Care<br>Grogan, S; Manchester Metropolitan University,<br>Chambers, Ruth; NHS Stoke on Trent,<br>Clark-Carter, David; Staffordshire University, Department of Psychology
**Primary Subject Heading**:	Public health
Secondary Subject Heading:	Health services research
Keywords:	PREVENTIVE MEDICINE, PRIMARY CARE, Coronary heart disease < CARDIOLOGY

SCHOLARONE™
Manuscripts

I, the Submitting Author has the right to grant and does grant on behalf of all authors of the Work (as defined in the below author licence), an exclusive licence and/or a non-exclusive licence for contributions from authors who are: i) UK Crown employees; ii) where BMJ has agreed a CC-BY licence shall apply, and/or iii) in accordance with the terms applicable for US Federal Government officers or employees acting as part of their official duties; on a worldwide, perpetual, irrevocable, royalty-free basis to BMJ Publishing Group Ltd ("BMJ") its licensees and where the relevant Journal is co-owned by BMJ to the co-owners of the Journal, to publish the Work in this journal and any other BMJ products and to exploit all rights, as set out in our licence.

The Submitting Author accepts and understands that any supply made under these terms is made by BMJ to the Submitting Author unless you are acting as an employee on behalf of your employer or a postgraduate student of an affiliated institution which is paying any applicable article publishing charge ("APC") for Open Access articles. Where the Submitting Author wishes to make the Work available on an Open Access basis (and intends to pay the relevant APC), the terms of reuse of such Open Access shall be governed by a Creative Commons licence – details of these licences and which Creative Commons licence will apply to this Work are set out in our licence referred to above.

Other than as permitted in any relevant BMJ Author's Self Archiving Policies, I confirm this Work has not been accepted for publication elsewhere, is not being considered for publication elsewhere and does not duplicate material already published. I confirm all authors consent to publication of this Work and authorise the granting of this licence.

For peer review only

Title

Quantitative examination of video-recorded NHS Health Checks: comparison of use of QRISK®2 versus JBS3 cardiovascular risk calculators

Authors

*Christopher J. Gidlow, Staffordshire University, Brindley Building, Leek Road, Stoke-on-Trent, ST4 2DF, c.gidlow@staffs.ac.uk

Naomi J. Ellis, Staffordshire University, Brindley Building, Leek Road, Stoke-on-Trent, ST4 2DF, n.j.ellis@staffs.ac.uk

Lisa Cowap, Staffordshire University, Brindley Building, Leek Road, Stoke-on-Trent, ST4 2DF, l.cowap@staffs.ac.uk

Victoria Riley, Staffordshire University, Brindley Building, Leek Road, Stoke-on-Trent, ST4 2DF, victoria.riley@staffs.ac.uk

Diane Crone, Cardiff Metropolitan University, Cyncoed Campus, Cyncoed Road, Cardiff, CF23 6XD, dmcrone@cardiffmet.ac.uk

Elizabeth Cottrell, Keele University, Keele, Newcastle-under-Lyme, ST5 5BG, e.cottrell@keele.ac.uk

Sarah Grogan, Manchester Metropolitan University, Manchester Campus, Bonsall Street, Manchester, M15 6GX, s.grogan@mmu.ac.uk

Ruth Chambers, Stoke-on-Trent Clinical Commissioning Group, Smithfield One Building, Stoke-on-Trent ST1 4FA, Ruth.Chambers@stoke.nhs.uk

David Clark-Carter, Staffordshire University, The Science Centre, Leek Road, Stoke-on-Trent, ST4 2DF, d.clark-carter@staffs.ac.uk

*Corresponding author

For peer review only

Abstract

Objectives

Quantitatively examine the content of NHS Health Check (NHSHC), patient-practitioner communication balance and differences when using QRISK®2 versus JBS3 CVD risk calculators.

Design

RIsk COmmunication (RICO) in NHS Health Check (NHSHC) is a qualitative study with quantitative process evaluation, comparing NHSHC using QRISK®2 or JBS3. We present data from the quantitative process evaluation.

Setting and participants

Twelve general practices in the West Midlands (England) conducted NHSHC using JBS3 or QRISK®2 (6/group). Patients were eligible for NHSHC based on national criteria (aged 40-74, no existing cardiovascular-related diagnoses, not taking statins). Recruitment was stratified by patient age, gender and ethnicity.

Methods

Video-recordings of NHSHC were coded, second-by-second, to quantify who was speaking and what was being discussed. Outcomes included consultation duration, practitioner verbal dominance [ratio of practitioner:patient speaking time (pr:pt ratio)], and proportion of time discussing CVD risk, risk factors and risk management.

Results

173 video-recorded NHSHC were analysed (73 QRISK, 100 JBS3). The sample was 51% female, 83% White British, with approximately equal proportions across age groups. NHSHC duration varied greatly (6.8-38.0 minutes). Most (60%) lasted less than 20 minutes. On average, CVD risk was discussed for less than two minutes (9.1±4.3% of consultation time). There were indications that, compared with NHSHC using JBS3, those with QRISK®2 involved less CVD risk discussion (JBS3 M=10.24, CI: 8.01-12.48% vs. QRISK2 M=7.44%, CI: 5.29-9.58) and were more verbally dominated by practitioners (pr:pt ratio JBS3 M=3.21%, CI: 2.44-3.97 vs. QRISK2=2.3%, CI: 1.89-2.81). The largest proportion of NHSHC time was spent discussing causal risk factors (M=37.54, CI: 32.92-42.17).

Conclusions

There was wide variation in NHSHC duration. Many were short and practitioner-dominated, with little time discussing CVD risk. JBS3 appears to extend CVD risk discussion and patient contribution. Qualitative examination of how it is used is necessary to fully understand the potential benefits of these differences.

Trial registration: ISRCTN ISRCTN10443908. Registered 7th February 2017.

Keywords: Cardiovascular disease; risk communication; Health Check; chronic disease prevention

For peer review only

Strengths and limitations of this study

- Video-recording of NHS Health Checks provide an objective record of practice across a range of general practices.
- Second-by-second coding was used to characterise the content of Health Checks that used either QRISK2 or JBS3 CVD risk.
- The study was limited to 12 general practices in the West Midlands of England
- Target sample size was not achieved.
- Qualitative data are necessary to provide further insight.

For peer review only

Introduction

Cardiovascular disease (CVD) accounts for over a quarter of UK deaths and costs the National Health Service (NHS) around £9 billion annually [1]. As CVD mortality decreases in the UK, there is a high prevalence of people living with CVD [2]. Prevention, therefore, remains a priority, for which the NHS Health Check (NHSHC) programme is an important part [3,4]. NHSHC aims to assess CVD risk factors among adults in England aged 40-74 years who are not known to have certain cardiovascular-related diseases [5]. It is the largest CVD risk identification and management programme of its kind globally, and has been linked with some increases in the detection of risk factors and chronic disease, and statin prescriptions [6,7], with mixed predictions of benefits from microsimulation studies [8,9].

NHSHC consultations typically take place in primary care with a primary care nurse, and comprise: (i) assessing patients' CVD risk, (ii) communication of CVD risk, which should inform (iii) discussion of CVD risk management through lifestyle or subsequent medical appointments or referrals. The NHSHC competence framework specifies that practitioners should understand CVD risk and be able to communicate to patients their CVD risk such that '*the client understands their level of risk*' [10, p21]. This should lead to discussion with the patient about management of that risk through '*person-centred conversations about their own reasons for change*' [10, p21], using techniques like motivational interviewing. This accords with the idea of shared decision-making, described as the '*pinnacle of patient-centred care*' [11, p780], whereby health professionals and individuals work together to agree management or support, based on evidence and the patient's preferences [12].

To date, little is known about the nature or content of real-world NHSHC consultations. Evidence regarding what takes place in the consultations is mainly limited to qualitative data from retrospective interviews with patients and practitioners asked to recall and reflect on their experiences [13]. Such data do have value. However, they do not present a complete understanding of the dynamics and interactions that can initiate subsequent actions or interventions (e.g., referral of patients to effective lifestyle support programmes, appropriate specialist referrals) that could lead to improved patient outcomes.

This paper reports the relative contributions of practitioner and patients, and the time spent discussing CVD risk and risk management in NHSHC consultations. This is important to understand as lack of time is the barrier to shared decision-making, most frequently cited by practitioners and patients [14]. Consultations in which the health professional and patient work together to identify risk management strategies, taking evidence and patient preference in to account [12], require more time than didactic encounters involving little patient involvement [14]. Studies of clinician-patient interactions have identified that short, clinician-dominated (or 'paternalistic') consultations, are less patient-centred and linked with low patient and clinician satisfaction [15–18], which in turn, have been linked with poorer patient outcomes, such as adherence to clinical recommendations and health-promoting behaviour [19,20]. While there is some evidence that brief interventions of just a few minutes can be contribute towards behaviour change [21], motivational interviewing, a technique recommended in NHSHC, may require 15 minutes or more to be effective [22]. Pieterse et al [14] summarised the relatively modest literature that specifically links time with shared decision-making. The authors noted that health professionals often feel under time pressure. Time with patients is constrained by a schedule that determines the maximum appointment length, and the need to complete all clinical and administrative tasks. Hurried practitioners might interrupt the conversation [23] or present information too quickly or using inappropriate language, reducing the likelihood that information will be retained [14]. For NHSHC, it is likely to be important that there is sufficient time

for a two-way interaction in which patients can be supported to develop their own CVD risk management strategy.

Time is also likely to be an important consideration in communication of CVD risk in NHSHC. Cypher et al. identified prerequisites of shared decision-making that included '*accurate, impartial, and comprehensible information*' presented by a practitioner who is '*proficient in communication and able to individualize data to a particular situation*' [24, p1]. All NHSHC must involve assessment of CVD risk using QRISK® (QRISK®2 and, more recently, QRISK®3) [25,26]. QRISK, which is embedded within primary care medical record software, provides a percentage risk of a CVD event in the next 10 years. This has to be communicated to patients for the NHSHC to be considered 'complete'. It is integrated within the general practice electronic medical record software, so can be calculated from pre-populated and new data. The score is then directly saved into the patient's record. However, there are limitations to the QRISK score and how it is used. First, 10-year risk estimates, such as those presented by QRISK have been criticised for being heavily influenced by age and gender, thereby underestimating risk in younger adults and women, and not accounting for risk from other diseases as effectively as long-term (lifetime) estimates [27,28]. Second, qualitative studies indicate limited practitioner and patient understanding of percentage CVD risk [13]. Practitioners report difficulties in explaining percentage CVD risk [29–32]. In turn, patients attending NHSHCs have been unable to recall being provided with a risk score or find it confusing [13].

In 2014, the Joint British Societies recommendations on the prevention of Cardiovascular Disease (JBS3) launched the JBS3 risk calculator. JBS3 has a primary focus on lifetime risk [27], but includes heart age [33–36] and uses multiple visual displays to present them (e.g., Cates plot, image of heart for heart age, visual analogue scales) [37]. It has also been designed to support communication of tailored information by allowing manipulation, and, thus demonstration of, the effects of risk factor modification (e.g., smoking cessation) on lifetime risk trajectory. There is some evidence that lifetime risk, as provided by JBS3, can identify raised CVD risk in some people that would not be picked up through conventional 10-year risk estimates (given within QRISK2) [38]; that heart age is more easily communicated to, and recalled by, patients [39,40]; and that graphical displays can be preferable for promoting risk-reducing behaviour [41]. Collectively, these attributes of JBS3 might accommodate a more extensive and higher quality interaction between patients and practitioners [33,37]. However, a comparison of the relative benefits JBS3 and QRISK®2 for communicating CVD risk in NHSHC has not been undertaken. Given that practitioners currently have choices in CVD risk calculators, establishing their relative values is important, particularly with regards to knowing which approach best promotes communication that supports positive behaviour change.

In summary, NHSHC aims to assess CVD risk and to prompt minimisation of risks identified. To date, there is insufficient knowledge about how NHSHC are conducted, the time spent discussing CVD risk and its management, and the potential of alternative CVD risk calculators, like JBS3. The RIsk COmmunication in NHS Health Check (RICO) is a large study of practitioner and patient perceptions and understanding of CVD risk when using the JBS3 or QRISK®2 CVD risk calculators. Video-recording methods in RICO have provided the first objective data of real-world NHSHC, allowing for extensive qualitative and quantitative analysis. In this paper we present the quantitative data, with three aims: (i) examine the time spent discussing CVD risk, risk factors and management in NHSHC consultations; (ii) explore the level of patient-practitioner communication balance; (iii) compare i and ii when NHSHC are conducted using QRISK®2 versus JBS3 CVD risk calculators.

Method

Study design

The RICO study is a qualitative study with quantitative process evaluation. A detailed description of the overall study, including sampling, recruitment and data collection is available [38]. Here, we report a quantitative comparison of the content of video-recorded NHSHC consultations.

The RICO protocol was approved the Health Authority Approval (11/11/2017) and the London-Dulwich Research Ethics Committee (11/09/17, ref: 17/LO/1463). Written informed consent was obtained for all participants prior to taking part.

Public and Public Involvement and Engagement

Our approach was informed by extensive Patient and Public Involvement and Engagement (PPIE). Engagement with Patient Participation Groups at three general practices on two occasions was used to gather opinion on the study concept and overall design, and subsequently, the methods and protocols. quality, participant consent and debrief processes and for development of the coding framework. Four mock NHSHC were used to test protocols, such as camera placement, video-recording. Two patient representatives sit on the Study Steering Committee for ongoing involvement of patients in project management and a virtual study patient group was established using a closed Facebook group (>260 members) for ongoing PPIE.

Setting and general practice recruitment

Data were collected across 12 general practices in the West Midlands of England that already delivered NHSHC (January 2017 to February 2019). Practices were recruited via the local Clinical Research Network (CRN). Six practice pairs, approximately matched by deprivation, were randomly assigned to one of two groups: QRISK®2 (usual practice) - practitioners continue to use QRISK®2 to calculate CVD risk; JBS3 (intervention) - practitioners calculate the CVD risk using JBS3 following brief training about the platform (no training regarding risk communication was provided). To enable assessment of differences arising from JBS3, compared to QRISK®2, as a minimum, those using JBS3 were asked to use the output screens showing: (i) heart age - estimate of heart age compared with someone of the same gender, ethnicity and risk factors at optimal levels; (ii) Healthy Years – estimate of the age the patient can expect to reach without a CVD event. They were also asked to show patients the effect of intervention on one or more of the CVD risk metrics (e.g., effect of smoking cessation on event-free survival age). A brief training video was created to guide clinicians (https://www.youtube.com/watch?v=idecGzlwIc4&feature=youtu.be).

Participants and recruitment

Patients were those eligible for NHSHC based on national criteria. Thus people were excluded if they were outside of the target age range (40-74 years), had existing diagnoses for certain cardiovascular-related chronic conditions, took statins, had received an NHSHC in the last five years, or were known to be at high risk of CVD [42]. Within each practice, the list of eligible patients was stratified according to age, gender and ethnicity, and invitations were sent to a representative sample. Postal invitations were distributed and follow-up calls were made by practice staff.

For peer review only

Practitioners all worked within primary care (8 Health Care Assistants, 6 Practice Nurses, 1 Sister) and already undertook NHSHC as part of routine practice. The only exception was one HCA who was new to NHSHC delivery.

Data sources and processing

Participating practices were asked to video-record NHSHCs using the allocated CVD risk calculator until 20 useable consultations were recorded (with written permission from patients and practice consent). Video-recorded NHSHC consultations were the main data source. Recorded NHSHC were viewed by two authors (LC and VR) and the content of consultations was characterised using a second-by-second coding framework developed specifically for this study. The framework comprised 36 items grouped in to six categories: patient-practitioner communication, Health Check general (e.g., collecting and inputting data), risk dialogue (e.g., overall discussion of risk, 10-year risk score reference, heart age, patient question on CVD risk), causal CVD risk factors (medical, lifestyle), risk management - lifestyle intervention, and risk management - medical intervention (Supplementary file 1). This allowed derivation of aggregate indicators for each consultation, to allow between-group comparisons:

- Length of NHSHC
- Time (absolute and proportion of consultation) discussing CVD risk, CVD risk factors (overall, lifestyle, medical), and risk management (lifestyle, medical)
- Practitioner dominance (ratio of practitioner: patient speaking time)
- Number and proportion of patients asking questions about CVD risk
- Use of heart age, Healthy Years (event-free survival age) and risk score manipulation (as fidelity check in the JBS3 group).

As noted previously [43], the coding process and framework development was iterative, using four mock NHSHC that were video-recorded as part of PPIE. To reach consistency in approach, two authors (NE and LC) coded mock NHSHC by consensus. Author VR then coded the same four consultations independently and Intraclass Correlation Coefficients (ICCs) demonstrated excellent inter-rater reliability (ICCs from .968 to .995). Once data collection had begun, NHSHC were coded by Authors LC and VR, with verification of 10% (two in every 20 independently coded) to mitigate the risk of coder drift throughout the study. ICCs ranged from .992 to .999, indicating excellent inter-rater reliability.

Data were extracted from patient medical records on patient sex, age and ethnic background [classified as White British (WBRI) or Black Minority Ethnic (BAME)].

Sample size

The target sample size was 240 (120 per group). A sample size calculation undertaken for the desired between-group quantitative comparison estimated that 120 consultations per group, six clusters per group with a two-tailed probability and alpha of .05, would provide statistical power of .8 to detect an effect size (r) = .24 (small to medium effect). The overall number of eligible practices from which the clusters were chosen was 625.

Statistical methods

Following checks for normal distributions, QRISK®2 and JBS3 groups were compared according to key variables. Confidence intervals (95%) were calculated taking account of the nature of the sampling which was in clusters; as usual, where the confidence intervals of the two groups do not overlap then the groups can be considered to differ significantly. Data processing and analysis were performed in SPSS 26.

Results

Participants

175 video-recorded NHSHC were completed, of which 173 were included in analysis (QRISK=73; JBS3=100; 2 excluded for practitioner process error that invalidated the consultation). The sample comprised approximately equivalent proportions of males and females, and proportions in White British versus BAME ethnic groups that were representative of the geographical region (Table 1). Average age of the JBS3 group (M=60.87; CI: 58.91-62.83) was higher than QRISK®2 (M=54.70, CI: 51.66-57.70), while 10-year CVD risks was slightly higher in the JBS3 group (M=9.71, CI: 7.85-11.57) than the QRISK®2 group (M=8.69, CI: 5.56-11.81).

Table 1. Participant characteristics

		Total		JBS3		QRISK		Difference
		n	%	n	%	n	%	r [c]
Age	40-54	60	34.68	24	24.00	36	49.32	.32
	55-64	54	31.21	30	30.00	24	32.88	
	65-74	59	34.10	46	46.00	13	17.81	
	Total	173		100		73		
Gender	Male	86	49.71	49	49.00	37	50.68	.05
	Female	87	50.29	51	51.00	36	49.32	
	Total	173		100		73		
Ethnicity[a]	WBRI	144	83.24	81	81.00	63	86.30	.07
	BAME	29	16.76	19	19.00	10	13.70	
	Total	173		100		73		
CVD risk category[b]	Low	104	60.12	57	57.00	47	64.38	.06
	Medium-High	67	38.73	41	41.00	26	35.62	
	Total	171		98		73		

[a] Ethnicity: WBRI, White British; BAME, Black and minority ethnic groups
[b] CVD risk categories: low is 10%, medium-high is ≥10%
[c] Effect size, r: where .1 is small, .3 is medium, .5 is large

Length of NHS Health Check consultations

Table 2 summarises the characteristics of NHSHC consultations by group, and overall. There was a wide range in consultation length, from just 6.8 to over 38 minutes, but the majority were between

15 and 20 minutes (Figure 1). Consultations were only slightly shorter on average in the QRISK®2 compared with JBS3 group (with a relatively small effect size of .13).

For peer review only

Table 2. Between-group comparisons of characteristics of NHSHC consultation

	Total Mean (95% CI)	SD	JBS3 Mean (95% CI)	SD	QRISK Mean (95% CI)	SD	Difference[e] (QRISK vs. JBS3) r
**Duration** (minutes)	20.06 (18.87, 21.24)	6.21	20.66 (18.89, 22.42)	5.65	19.24 (15.28, 23.19)	6.84	.13
**Verbal dominance**							
% of total time practitioner speaking	50.07 (45.90, 54.24)	9.55	46.60 (41.36, 51.84)	8.79	54.82 (50.00, 59.64)	8.48	.42
% of total time patient speaking	23.37 (19.87, 26.87)	10.62	24.67 (20.47, 28.87)	10.53	21.6 (15.71, 27.43)	10.56	.15
Verbal dominance ratio[a]	2.70 (2.23, 3.19)	1.50	2.35 (1.89, 2.81)	1.31	3.21 (2.44, 3.97)	1.62	.27
**% of total HC time:**							
Discussing CVD risk	9.06 (7.36, 10.76)	4.30	10.24 (8.01, 12.48)	4.07	7.44 (5.29, 9.58)	4.08	-.32
Discussing risk factors							
Total	37.54 (32.92, 42.17)	12.96	35.33 (27.76, 42.90)	13.29	40.58 (36.20, 44.96)	11.91	.22
Medical[b]	21.34 (18.35, 24.33)	9.41	20.13 (15.33, 24.94)	9.38	22.98 (19.96, 26.31)	9.26	.16
Lifestyle[c]	16.11 (13.79, 18.44)	7.03	15.08 (11.87, 18.30)	6.49	17.52 (14.16, 20.88)	7.53	.16
Discussing risk management							
Total	19.64 (16.48, 22.81)	11.37	18.82 (13.92, 23.73)	11.12	20.77 (16.59, 24.94)	11.69	.08
Lifestyle	16.59 (13.44, 19.74)	10.2	15.94 (11.27, 20.62)	10.04	17.48 (12.95, 22.01)	10.41	.08
Medical[d]	63%		58%		69.9%		-.12

[a] Ratio of practitioner: patient talking (where higher value indicates greater practitioner verbal dominance);
[b] Includes medical history, family history, weight, blood pressure, cholesterol, diabetes, mental health and well-being;
[c] Includes diet, alcohol, physical activity, smoking
[d] Percentage of patients with whom medical interventions were discussed
[e] Effect size, r: where .1 is small, .3 is medium, .5 is large

For peer review only

Discussion of CVD risk

Overall, less than 10% of overall consultations time was devoted to CVD risk discussion, which equated to 1.7±0.83 minutes. A higher proportion of consultation time was spent discussing CVD risk using JBS3 (equivalent to 2.1±0.82 minutes) compared with QRISK®2 (equivalent to 1.31±0.63 minutes), with a medium effect size. Nearly all NHSHC in both groups included reference to the 10-year % CVD risk score (94 vs. 94.5%, r = .01). The proportion of patients asking questions about CVD risk was higher in the JBS3 versus QRISK®2 group (32.0 vs. 12.3%, r=.23).

Within the JBS3 group, nearly all practitioners discussed heart age (100%) and healthy years (97%), and manipulated the risk score(s) to show the potential effect of intervention on risk (92%). This showed fidelity to the requested minimum use of JBS3 outputs. Use of heart age and risk manipulation were also evident in 52.1% and 21.9% of QRISK®2 consultations, respectively. This is a result of two general practices in the QRISK®2 group using Informatica (a software addition that offers some JBS3 functionalities), and because heart age and allows manipulation are possible (but not main features) in QRISK®2.

Discussion of CVD risk factors and risk management

Over one-third of total NHSHC time was spent discussing CVD risk factors. This was slightly higher in the QRISK®2 versus JBS3 informed NHSHCs, but with wide variation within groups (Table 2).

Interventions to manage risk were discussed for approximately one-fifth of total consultation time and predominantly related to lifestyle, rather than medical intervention, where just over 30% of QRISK®2 and 42% of JBS3 did not mention it (r=-.12).

Verbal dominance

Practitioners spoke for just over half of total time in QRISK®2 consultations and just under half in JBS3 (Figure 2). There was an indication of higher practitioner verbal dominance in NHSHC using QRISK®2 versus JBS3 consultations (r=.27).

Discussion

Main findings

We present the first objective data on the content of NHSHC consultations, with comparison of QRISK®2 and JBS3 CVD risk calculators. Second-by-second coding of 173 video-recorded NHSHC consultations from 12 general practices provided new insights regarding duration, verbal dominance and allocation of time to different components of the consultation. Main findings in relation to our study aims were as follows. First, the length of NHSHC consultations was varied and often short (most <20 minutes). Less than 10% of time (<2 minutes) was spent discussing the individual's calculated CVD risk, and the largest component was discussion of causal CVD risk factors. Second, there was evidence of practitioner verbal dominance as, on average, practitioners spoke for half of total consultation time (compared with ~23% with patients speaking). Third, compared with QRISK2, there were indications that use of JBS3 was associated with more discussion of CVD risk, less discussion of CVD risk factors,

For peer review only

reduced practitioner verbal dominance and, on average, required only 1.4 additional minutes (<90 seconds).

What this study adds

The variable and often short duration of NHSHC, combined with practitioner verbal dominance are a potential concern. Sixty per cent of NHSHC in the RICO study lasted less than 20 minutes, 18% less than 15 minutes, and 4% were under 10 minutes. Nearly all (95%) had a verbal dominance ratio greater than one, 57% had a ratio of two or more, and 36% were over three, which indicates some degree of verbal dominance. Practitioner verbal dominance suggests more information provision than the preferred patient-centred two-way interaction that seeks to understand the patients' contexts, priorities and preferences and goal-setting. While information provision is an important part of the process, the literature on clinician-patient interactions suggests that short, paternalistic primary care consultations are associated with low patient and clinician satisfaction [18]. This can have negative implications for patient outcomes, potentially reducing adherence to clinician recommendations, ability to self-care or make informed decision about their health [15–17].

Practitioner-patient interactions are complex [44]. The practitioner-patient balance has been discussed as a spectrum of locus of control, with a paternalistic health professional at one end and the 'informed patient' at the other [45]. The latter requires that control is mutual or exchanged, allowing a 'negotiated plan'; in this case, a negotiated plan to manage CVD risk. The nature of interactions have been conceptualised and measured variously. For example, 'patient enablement', whereby patients have understanding, confidence and coping ability following enabling consultations [15], and 'physician–patient collaboration', where both physician and patient are active participants in consultations [46]. A common feature of consultations that foster these beneficial relationships (and should lead to better outcomes) is the active participation of patients in the consultation and decisions about their health care. Our data indicate that there is scope in NHSHC for greater patient participation, especially as motivational interviewing should be a key feature of NHSHC and relies on a patient-centred approach [25].

The length of appointments that practices allocated to NHSHC ranged from 15 to 30 minutes. This showed variation in the time that general practices were willing to give to NHSHC, and the associated time constraints for practitioners deliver NHSHC in those practices. Yet, video-recordings highlighted even greater variability in the actual duration of practitioner-patient interaction (6.8 to 38 minutes). The length of primary care nurse appointments varies by appointment type. NHSHCs require a number of clinical and administrative tasks, such as: CVD risk assessment involving measurement of (and entry of data for) weight, blood pressure and, sometimes, cholesterol through point-of-care testing lifestyle; assessment (physical activity, alcohol, diet); explaining to patients their CVD risk score(s) and what it means; patient-centred discussion of risk management to prompt risk-reducing behaviours. In light of evidence that shared decision-making takes time and techniques like motivational interviewing might require 15 or more minutes to be effective, it is reasonable to suggest that consultations lasting much less than 20 minutes are unlikely to achieve all of the above while allowing time for appropriate levels of patient participation as part of a mutual exchange.

A primary focus of the RICO project is CVD risk communication in NHSHC. To promote health-protective behaviours that reduce CVD risk, practitioners need to understand the risk information and be able to communicate it effectively such that patients leave the consultation with the knowledge and intention to act [41]. The little time dedicated to CVD risk discussion (<10%), especially when using

For peer review only

QRISK2, accords with findings from the 2017 systematic review of qualitative data studies [47]. The review authors identified patients' 'limited understanding of the risk score' as a common theme. Some patients reported improved understanding of CVD risk following their NHSHC, but many did not recall being told their risk, found it confusing or had misinterpreted their risk score.

Our data confirmed that nearly all practitioners using JBS3 conveyed heart age, healthy years and used risk score manipulation (as requested). This appeared to result in a slightly higher proportion of that time spent on CVD risk discussion in JBS3-informed consultations (although not reaching significance), but equated to less than one additional minute on average. This raises the question of whether JBS3 does help to engender better patient engagement and understanding of their risk, or simply more information provision. That one in three patients in JBS3 consultations asked questions about their CVD risk (32%), compared with one in eight QRISK®2 patients (12%), suggests better engagement, but qualitative analysis is required to provide a more complete understanding. RICO did not include extensive practitioner training in use of JBS3. Rather, an introductory briefing with supporting DVD were used to maintain the ecological validity of exploring how practitioners would use JBS3 if made available in practice (not the effect of additional training, which is generally not available in practice) [48]. Such training might be required and general CVD risk communication training for NHSHC practitioners has shown some benefits for practitioner confidence and understanding [48].

The apparent focus on causal risk factors, discussion of which comprised 38% of NHSHC time overall (over 40% when using QRISK2), is perhaps an indication that practitioners did not spend much time explaining and inviting discussion around the risk score(s) and what they mean, but focused more on the potential causes and their management. This could be interpreted positively as a solution-focused approach that focuses on the tangible factors that might be easily understood; i.e., how their medical history and lifestyle can lead and help to prevent CVD. However, it is important that this discussion is in the context of the patient's CVD risk and tailored to their lifestyle and priorities. Risk communication is challenging [37]. If delivered effectively, it can enhance knowledge and decision-making about treatment, and can empower and create autonomy [49]. Conversely, poor communication of risk can cause patients anxiety and reduce confidence in health professionals, or may result in the perception that action is futile [50]. Therefore, the 38% and 20% of NHSHC time spent discussing CVD risk factors and management, respectively, could be undermined if the patient is confused or alarmed by the preceding information about their CVD risk.

Implications for practice

The 2019 green paper, *Advancing our health: prevention in the 2020s*, set out plans for an evidence-based review of NHSHC to maximise benefit in the next decade (18). This indicates a future for NHSHC, with evidence-based changes to delivery. Despite the importance of risk communication, associated recommendations in NHSHC best practice guidance [25] and the practitioner competence framework that includes CVD risk communication [10], ours are the first objective data showing the varied and often short NHSHC, the limited time devoted to CVD risk discussion, and the potential for JBS3 to enhance this. We know that training in NHSHC is a challenge to optimal implementation [13]. Practitioners generally receive little (or no) training in CVD risk communication for NHSHC, and can lack the associated confidence and skills [48]. Our data strengthen the case for NHSHC practitioner training, ideally with patient involvement, to redress the practitioner verbal dominance and help to make the discussion of CVD risk appropriate and positive for patients. JBS3 (or similar tools) could form part of this solution.

For peer review only

Strengths and limitations

The strengths of this study include video-recording of NHSHC across a diverse range practices stratified by deprivation, stratified sampling of patients, and our comprehensive coding framework with excellent inter-rater reliability that offers a methodological contribution. A number of limitations are recognised. First, challenges with practice and patient recruitment meant that we did not achieve our target sample size of 240 (120/group). A larger sample size might have identified more marked between-group differences through increasing the precision of our estimates (i.e., narrowing confidence intervals). Second, there was a between-group difference in mean age. This did not translate into a difference in CVD risk, which would have introduced more systematic bias into the sample. But it would mean that risk was underestimated in larger proportion of younger people in the QRISK®2 group (compared with the JBS3 group). Third, these quantitative data do not tell us directly about quality of discussion or exact nature of patient response. However, the duration of time spent on different elements of risk communication and subsequent management planning do provide valuable indirect evidence about patient engagement and balance of contributions and content within NHCHC consultations. Fourth, we cannot claim that our results are generalizable to the rest of England. However, we have a good balance of males/females, a good age range and proportions of WBRI and BAME ethnic groups that were appropriate for the region. Fourth, after commencing data collection, we discovered that two QRISK®2 practices used Informatica, an addition to practice software that has some of the JBS3 functionalities. To maintain the ecological validity of a 'usual care' group, the 34 patients from these practices were retained, as they will be for qualitative analysis. Finally, it is possible that being video-recorded affected practitioner behaviour (Hawthorne effect). To mitigate this, our PPIE explored camera position to best capture patient response and minimise practitioner awareness of the camera. Practitioners in mock NHSHC reported forgetting about the camera during consultations. The Hawthorne effect tends to improve performance [51], which would mean that any effect would lead to an underestimate of issues with NHSHC delivery.

Conclusions

Duration of the practitioner-patient interaction in NHSHC is varied and, in many cases, short. Our data highlight that little NHSHC time is devoted to discussing the patients' calculated CVD risk, however it does appear that JBS3 can support an extended discussion and might prompt more patient engagement. The impact on practitioner verbal dominance on patient experience and outcomes should be further explored. Public Health England's competence framework for NHSHC specifies the need for NHSHC practitioners to be trained in communicating the risk scores and to engage in person-centred conversations around risk-reducing behaviour changes [10]. Although our qualitative findings will help to provide a more complete picture and elucidate the true potential of JBS3, our current data support the need for practitioner training in this area and, potentially, for additions to standard competencies within health professional training.

For peer review only

Authors' contributions

CG, DC, NE, EC, SG and RC contributed to the conceptualisation and design. NE, LC and CG led the development of the coding system and DCC advised on verification testing. LC and VR completed coding of recorded NHSHC. NE, LC and VR contributed to the Patient Public Involvement. DCC completed statistical analysis. CG led manuscript preparation. All authors contributed to the writing of the manuscript. All authors read and approved the final manuscript.

Competing interests

The authors declare that they have no competing interests.

Funding

This project is funded by the National Institute for Health Research HTA Programme (project number 15/170/02). The funders had no role in study design, data collection and analysis, decision to publish, or preparation of the manuscript.

Data availability

No additional data are available.

Ethics approval and consent to participate

The study was approved by the Health Authority Approval (11/11/2017) and the London - Dulwich Research Ethics Committee (11/09/17) (reference: 17/LO/1463). Written informed consent will be obtained for all participants prior to taking part.

Acknowledgements

The authors would like to acknowledge Clinical Research Network West Midlands colleagues for advice in developing protocols, and members of the PPG and the virtual PPI participants who have informed study development.

This report paper independent research funded by the National Institute for Health Research (NIHR). The views expressed are those of the author and not necessarily those of the NHS, the NIHR or the Department of Health and Social Care.

For peer review only

References

1 Wilkins E, L. W, Wickramasinghe K, *et al. European Cardiovascular Disease Statistics 2017 edition*. Brussels: European Heart Network 2017.

2 Bhatnagar P, Wickramasinghe K, Wilkins E, *et al.* Trends in the epidemiology of cardiovascular disease in the UK. *Heart* 2016;**102**:1945–52. doi:10.1136/heartjnl-2016-309573

3 National Health Service. *The NHS Long Term Plan*. London: NHS 2019.

4 Department of Health and Social Care. *Advancing our health: prevention in the 2020s – consultation document*. London: HMSO 2019.

5 Department of Health. *Putting prevention first. NHS Health Check: vascular risk assessment and management best practice guidance*. London: Department of Health 2009.

6 Robson J, Dostal I, Sheikh A. The NHS health check in England: an evaluation of the first 4 years. *BMJ Open* 2016;**6**. doi:10.1136/bmjopen-2015-008840

7 Forster AS, Burgess C, Dodhia H, *et al.* Do health checks improve risk factor detection in primary care? Matched cohort study using electronic health records. *J Public Health* 2016;**38**:552–9. doi:10.1093/pubmed/fdv119

8 Mytton OT, Jackson C, Steinacher A, *et al.* The current and potential health benefits of the National Health Service Health Check cardiovascular disease prevention programme in England: A microsimulation study. *PLoS Med* 2018;**15**:e1002517. doi:10.1371/journal.pmed.1002517

9 Kypridemos C, Allen K, Hickey GL, *et al.* Cardiovascular screening to reduce the burden from cardiovascular disease: microsimulation study to quantify policy options. *BMJ* 2016;**353**::i2793. doi:10.1136/bmj.i2793

10 Public Health England. *NHS Health Check competence framework*. London: PHE 2015.

11 Barry MJ, Edgman-Levitan S. Shared Decision Making — The Pinnacle of Patient-Centered Care. *N Engl J Med* 2012;**366**:780–1. doi:10.1056/NEJMp1109283

12 National Health Service. Shared decision making. 2019.https://www.england.nhs.uk/shared-decision-making/ (accessed 20 Nov 2019).

13 Usher-Smith JA, Mant J, Martin A, *et al. NHS Health Check Programme Rapid Evidence Synthesis*. Cambridge: The Primary Care Unit, University of Cambridge; RAND Europe 2017.

14 Pieterse AH, Stiggelbout AM, Montori VM. Shared Decision Making and the Importance of Time. *JAMA* 2019;**322**:25–6. doi:10.1001/jama.2019.3785

15 Pawlikowska T, Zhang W, Griffiths F, *et al.* Verbal and non-verbal behavior of doctors and patients in primary care consultations - How this relates to patient enablement. *Patient Educ Couns* 2012;**86**:70–6. doi:10.1016/j.pec.2011.04.019

16 G Howie J, Heaney D, Maxwell M. Measuring quality in general practice. Pilot study of a needs, process and outcome measure. *Occas Pap R Coll Gen Pract* 1997;**75**:i–xii, 1.

17 Street RL, Makoul G, Arora NK, *et al.* How does communication heal? Pathways linking clinician–patient communication to health outcomes. *Patient Educ Couns* 2009;**74**:295–301. doi:https://doi.org/10.1016/j.pec.2008.11.015

18 Roter D, Stewart M, Putnam S, *et al.* Communication patterns of primary care physicians. *JAMA* 1997;**277**:350–6. doi:10.1001/jama.1997.03540280088045

For peer review only

19 Doyle C, Lennox L, Bell D. A systematic review of evidence on the links between patient experience and clinical safety and effectiveness. *BMJ Open* 2013;**3**. doi:10.1136/bmjopen-2012-001570

20 Stacey D, Légaré F, Lewis K, *et al.* Decision aids for people facing health treatment or screening decisions. Issue 4. Art. No.: CD001431. DOI: 10.1002/14651858.CD001431.pub5. *Cochrane Database Syst Rev* 2017.

21 National Institute of Health and Care Excellence. *Public Health Draft Guidance: Behaviour Change*. London: NICE 2017.

22 Rubak S, Sandboek A, Lauritzen T, *et al.* Motivational interviewing: a systematic review and meta-analysis. *Br J Gen Pr* 2005;**55**:305–12.

23 Singh Ospina N, Phillips KA, Rodriguez-Gutierrez R, *et al.* Eliciting the Patient's Agenda-Secondary Analysis of Recorded Clinical Encounters. *J Gen Intern Med* 2019;**34**:36–40. doi:10.1007/s11606-018-4540-5

24 Cypher RL. Shared Decision-Making: A Model for Effective Communication and Patient Satisfaction. *J Perinat Neonatal Nurs* 2019;**33**.

25 Public Health England. *NHS Health Check Best practice guidance*. London: Public Health England 2017.

26 National Institutes of Health and Care Excellence. *Cardiovascular disease: risk assessment and reduction, including lipid modification. Clinical guideline [CG181]*. London: NICE 2015.

27 JBS3 Board. Joint British Societies' consensus recommendations for the prevention of cardiovascular disease (JBS3). *Heart* 2014;**100**:ii1–67. doi:10.1136/heartjnl-2014-305693

28 Karmali KN, Lloyd-Jones DM. Adding a life-course perspective to cardiovascular-risk communication. *Nat Rev Cardiol* 2013;**10**:111–5. doi:10.1038/nrcardio.2012.185

29 Gigerenzer G, Hoffrage U. How to improve Bayesian reasoning without instruction: Frequency formats. *Psychol Rev* 1995;**102**:684–704.

30 Hoffrage U, Gigerenzer G. Using natural frequencies to improve diagnostic inferences. *Acad Med* 1998;**73**:538–40. doi:10.1097/00001888-199805000-00024

31 Gigerenzer G, Edwards A. Simple tools for understanding risks: from innumeracy to insight. *BMJ Br Med J* 2003;**327**:741–4. doi:10.1136/bmj.327.7417.741

32 Slovic P, Monahan J, MacGregor DG. Violence risk assessment and risk communication: The effects of using actual cases, providing instruction, and employing probability versus frequency formats. *Law Hum Behav* 2000;**24**:271–96. doi:10.1023/A:1005595519944

33 Wells S, Kerr A, Broadbent E, *et al.* Does Your Heart Forecast help practitioner understanding and confidence with cardiovascular disease risk communication? *J Prim Heal Care* 2011;**3**:4–9.

34 Bonner C, Jansen J, Newell BR, *et al.* I Don't Believe It, But I'd Better Do Something About It: Patient Experiences of Online Heart Age Risk Calculators. *J Med Internet Res* 2014;**16**.

35 Soureti A, Hurling R, Murray P, *et al.* Evaluation of a cardiovascular disease risk assessment tool for the promotion of healthier lifestyles. *Eur J Cardiovasc Prev Rehabil* 2010;**17**:519–23.

36 Reed JL, Prince SA, Cole CA, *et al.* Workplace physical activity interventions and moderate-to-vigorous intensity physical activity levels among working-age women: a systematic review protocol. *Syst Rev* 2014;**3**:147. doi:10.1186/2046-4053-3-147 [doi]

For peer review only

37 Spiegelhalter D, Pearson M, Short I. Visualizing uncertainty about the future. *Science (80-)* 2011;**333**:1393–400.

38 Johns I, Moschonas KE, Medina J, *et al.* Risk classification in primary prevention of CVD according to QRISK2 and JBS3 {\textquoteleft}heart age{\textquoteright}, and prevalence of elevated high-sensitivity C reactive protein in the UK cohort of the EURIKA study. *Open Hear* 2018;**5**. doi:10.1136/openhrt-2018-000849

39 Hawking MKD, Timmis A, Wilkins F, *et al.* Improving cardiovascular disease risk communication in NHS Health Checks: a qualitative study. *BMJ Open* 2019;**9**. doi:10.1136/bmjopen-2018-026058

40 Bonner C, Bell K, Jansen J, *et al.* Should heart age calculators be used alongside absolute cardiovascular disease risk assessment? *BMC Cardiovasc Disord* 2018;**18**:19. doi:10.1186/s12872-018-0760-1

41 Waldron C-A, van der Weijden T, Ludt S, *et al.* What are effective strategies to communicate cardiovascular risk information to patients? A systematic review. *Patient Educ Couns* 2011;**82**:169–81. doi:http://dx.doi.org/10.1016/j.pec.2010.04.014

42 Public Health England. *NHS Health Check Programme: Best practice guidance*. London: PHE 2013.

43 Gidlow CJ, Ellis NJ, Cowap L, *et al.* A qualitative study of cardiovascular disease risk communication in NHS Health Check using different risk calculators: protocol for the RIsk COmmunication in NHS Health Check (RICO) study. *BMC Fam Pract* 2019;**20**:11. doi:10.1186/s12875-018-0897-0

44 Asan O, Montague E. Using video-based observation research methods in primary care health encounters to evaluate complex interactions. *Inform Prim Care* 2014;**21**:161—170. doi:10.14236/jhi.v21i4.72

45 Taylor K. Paternalism, participation and partnership—The evolution of patient centeredness in the consultation. *Patient Educ Couns* 2009;**74**:150–5. doi:https://doi.org/10.1016/j.pec.2008.08.017

46 Arbuthnott A, Sharpe D. The effect of physician–patient collaboration on patient adherence in non-psychiatric medicine. *Patient Educ Couns* 2009;**77**:60–7. doi:https://doi.org/10.1016/j.pec.2009.03.022

47 Usher-Smith JA, Harte E, Maclure C, *et al.* Patient experience of NHS health checks: A systematic review and qualitative synthesis. *BMJ Open* 2017;**7**. doi:10.1136/bmjopen-2017-017169

48 Riley VA, Gidlow CJ, Ellis NJ, *et al.* Improving Cardiovascular Disease Risk Communication in the UK National Health Service Health Check Programme. *Patient Educ Couns* 2019;**102**:2016–23. doi:https://doi.org/10.1016/j.pec.2019.05.014

49 Ahl AS, Acree JA, Gipson PS, *et al.* Standardization of nomenclature for animal health risk analysis. *Rev Sci Tech* 1993;**12**:1045–53.

50 Thomson R, Edwards A, Grey J. Risk communication in the clinical consultation. Clin. Med. J. R. Coll. Physicians London. 2005;**5**:465–9. doi:10.7861/clinmedicine.5-5-465

51 Yanes AF, McElroy LM, Abecassis ZA, *et al.* Observation for assessment of clinician performance: a narrative review. *BMJ Qual Saf* 2015;**25**:46–55. doi:10.1136/bmjqs-2015-004171

For peer review only

For peer review only

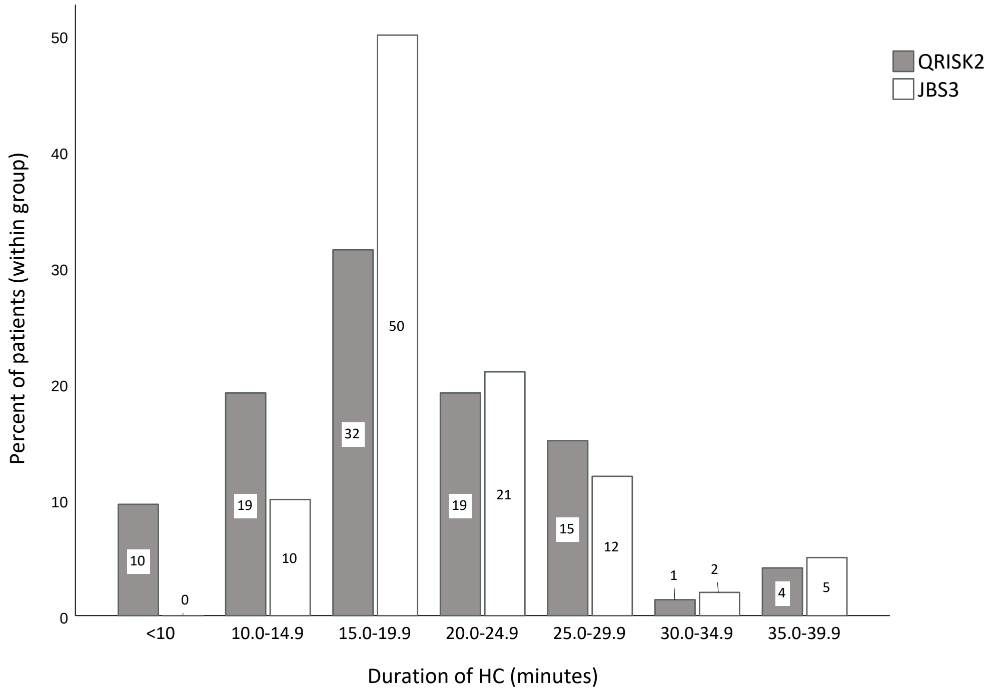

For peer review only - http://bmjopen.bmj.com/site/about/guidelines.xhtml

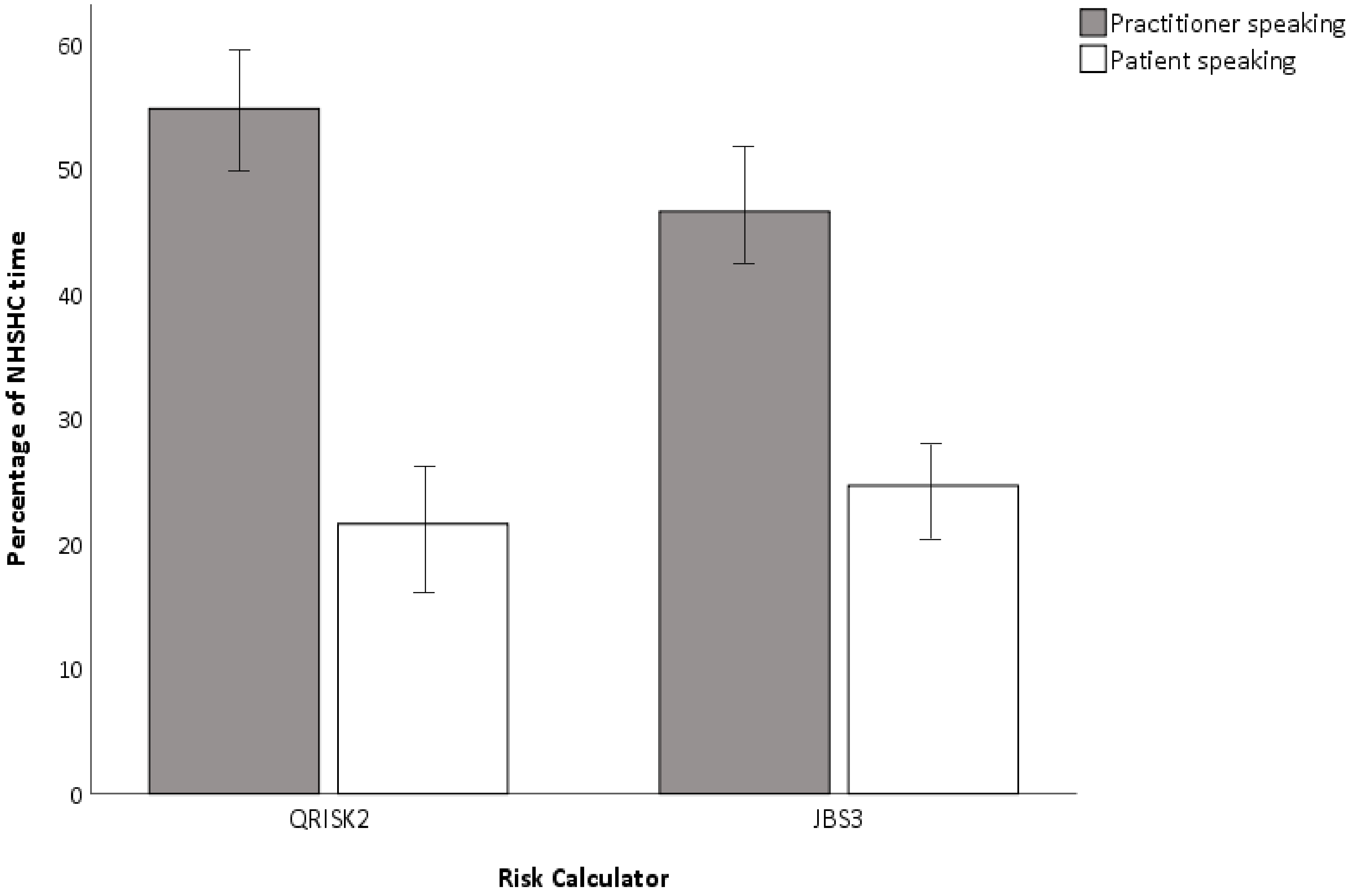

Error bars: 95%

Table S1. Coding framework data variables

Category	Code	Code Title
Patient-practitioner communication	PrDT	Practitioner dominated talk
	PtDT	Patient dominated talk
	NT	No talking
Health Check general	I2HC	Introduction to Health Check
	HCSUM	Health Check summary
	C&ID	Collecting and Inputting Data
	TRG	Test result/recording given
Risk dialogue	DoR	Discussion of Risk
	RSRef	Risk score reference
	HARef	Heart age reference
	SARef	Survival age free of cardiac event reference
	RSM	Risk score manipulation using either QRISK2 or JBS3
	CVDRQ	Patient question about CVD risk
Causal CVD risk factors	FH-MHIS	Risk factor discussion – medical history (Med)
	RF-FH	Risk factor discussion – family history (Med)
	RF-W	Risk factor discussion – weight (Med)
	RF-C	Risk factor discussion – cholesterol (Med)
	RF-BP	Risk factor discussion – blood pressure (Med)
	RF-D	Risk factor discussion – diabetes (Med)
	RF-MH&W	Risk factor discussion – mental health & wellbeing (Med)
	RF-PA	Risk factor discussion – physical activity/inactivity (LS)
	RF-DIET	Risk factor discussion – diet (LS)
	RF-A	Risk factor discussion – alcohol consumption (LS)
	RF-S	Risk factor discussion – smoking (LS)
Risk management - Lifestyle interventions	I-W	Intervention discussion - weight
	I-C	Intervention discussion – cholesterol
	I-BP	Intervention discussion – blood pressure
	I-D	Intervention discussion – diabetes
	I-MW&W	Intervention discussion – mental health & wellbeing
	I-PA	Intervention discussion – physical activity
	I-DIET	Intervention discussion – diet
	I-A	Intervention discussion – alcohol
	IL-S	Intervention discussion – smoking
Risk management - Medical interventions	I-GP	Intervention discussion – GP appointment
	I-PR	Intervention discussion – practitioner appointment (practice nurse or HCA)
	I-M	Intervention discussion - medication
Comments & Recommendations		

For peer review only

STROBE Statement—checklist of items that should be included in reports of observational studies

	Item No	Recommendation (page information is reported on)
**Title and abstract**	1	(*a*) Indicate the study's design with a commonly used term in the title or the abstract (p.2)
		(*b*) Provide in the abstract an informative and balanced summary of what was done and what was found (p.2)
**Introduction**		
Background/rationale	2	Explain the scientific background and rationale for the investigation being reported (p.4)
Objectives	3	State specific objectives, including any prespecified hypotheses (p.5)
**Methods**		
Study design	4	Present key elements of study design early in the paper (p.4)
Setting	5	Describe the setting, locations, and relevant dates, including periods of recruitment, exposure, follow-up, and data collection (p.6)
Participants	6	(*a*) *Cohort study*—Give the eligibility criteria, and the sources and methods of selection of participants. Describe methods of follow-up<br>*Case-control study*—Give the eligibility criteria, and the sources and methods of case ascertainment and control selection. Give the rationale for the choice of cases and controls<br>*Cross-sectional study*—Give the eligibility criteria, and the sources and methods of selection of participants (p.6)
		(*b*) *Cohort study*—For matched studies, give matching criteria and number of exposed and unexposed<br>*Case-control study*—For matched studies, give matching criteria and the number of controls per case
Variables	7	Clearly define all outcomes, exposures, predictors, potential confounders, and effect modifiers. Give diagnostic criteria, if applicable (p.6-7)
Data sources/ measurement	8*	For each variable of interest, give sources of data and details of methods of assessment (measurement). Describe comparability of assessment methods if there is more than one group (p.7)
Bias	9	Describe any efforts to address potential sources of bias (p.6, 10-11)
Study size	10	Explain how the study size was arrived at (p.7)
Quantitative variables	11	Explain how quantitative variables were handled in the analyses. If applicable, describe which groupings were chosen and why (p.6-7)
Statistical methods	12	(*a*) Describe all statistical methods, including those used to control for confounding (p.7)
		(*b*) Describe any methods used to examine subgroups and interactions (n/a)
		(*c*) Explain how missing data were addressed (n/a)
		(*d*) *Cohort study*—If applicable, explain how loss to follow-up was addressed<br>*Case-control study*—If applicable, explain how matching of cases and controls was addressed<br>*Cross-sectional study*—If applicable, describe analytical methods taking account of sampling strategy (n/a)
		(*e*) Describe any sensitivity analyses (p.11, Supplementary file, Table S2)

Continued on next page

For peer review only - http://bmjopen.bmj.com/site/about/guidelines.xhtml

**Results**		
Participants	13*	(a) Report numbers of individuals at each stage of study—eg numbers potentially eligible, examined for eligibility, confirmed eligible, included in the study, completing follow-up, and analysed (n/a)
		(b) Give reasons for non-participation at each stage
		(c) Consider use of a flow diagram
Descriptive data	14*	(a) Give characteristics of study participants (eg demographic, clinical, social) and information on exposures and potential confounders (Table 1)
		(b) Indicate number of participants with missing data for each variable of interest (n/a)
		(c) *Cohort study*—Summarise follow-up time (eg, average and total amount)
Outcome data	15*	*Cohort study*—Report numbers of outcome events or summary measures over time
		Case-control study—Report numbers in each exposure category, or summary measures of exposure (Table 1)
		Cross-sectional study—Report numbers of outcome events or summary measures
Main results	16	(*a*) Give unadjusted estimates and, if applicable, confounder-adjusted estimates and their precision (eg, 95% confidence interval). Make clear which confounders were adjusted for and why they were included (n/a)
		(*b*) Report category boundaries when continuous variables were categorized (Table 2)
		(*c*) If relevant, consider translating estimates of relative risk into absolute risk for a meaningful time period (n/a)
Other analyses	17	Report other analyses done—eg analyses of subgroups and interactions, and sensitivity analyses (n/a)
**Discussion**		
Key results	18	Summarise key results with reference to study objectives (p.8-9)
Limitations	19	Discuss limitations of the study, taking into account sources of potential bias or imprecision. Discuss both direction and magnitude of any potential bias (p.11)
Interpretation	20	Give a cautious overall interpretation of results considering objectives, limitations, multiplicity of analyses, results from similar studies, and other relevant evidence (p.9-11)
Generalisability	21	Discuss the generalisability (external validity) of the study results (p.11)
**Other information**		
Funding	22	Give the source of funding and the role of the funders for the present study and, if applicable, for the original study on which the present article is based (p.12)

*Give information separately for cases and controls in case-control studies and, if applicable, for exposed and unexposed groups in cohort and cross-sectional studies.

Note: An Explanation and Elaboration article discusses each checklist item and gives methodological background and published examples of transparent reporting. The STROBE checklist is best used in conjunction with this article (freely available on the Web sites of PLoS Medicine at http://www.plosmedicine.org/, Annals of Internal Medicine at http://www.annals.org/, and Epidemiology at http://www.epidem.com/). Information on the STROBE Initiative is available at www.strobe-statement.org.

For peer review only
